# Helmet (Health impact of e-bikes and e-scooters) study: Data collection methods and information gathered for the evaluation of the introduction of share hire schemes

**DOI:** 10.3310/nihropenres.13857.1

**Published:** 2025-05-07

**Authors:** Miranda EG Armstrong, James Garbutt, Tim Jones, Ben Spencer, Ian Philips, Sabina Sanghera, Lesley Welch, Rayne Roberts, Frank de Vocht, Russell Jago, Ruth Salway

**Affiliations:** 1 Centre for Exercise, Nutrition and Health Sciences, University of Bristol School for Policy Studies, Bristol, England, BS8 1TZ, UK; 2 NIHR Bristol Biomedical Research Centre, University Hospitals Bristol and Weston NHS Foundation Trust and University of Bristol, Bristol, UK; 3 School of the Built Environment, Oxford Brookes University School of the Built Environment, Oxford, England, OX3 0BP, UK; 4 Institute for Transport Studies, University of Leeds Institute for Transport Studies, Leeds, England, LS2 9JT, UK; 5 Health Economics Bristol (HEB), Population Health Sciences, University of Bristol Medical School, Bristol, England, BS8 1NU, UK; 6 Patient and Public Involvement, Bristol, BS8 1QU, UK; 7 Centre for Public Health, Population Health Sciences, University of Bristol Medical School, Bristol, England, BS8 1UD, UK; 8 NIHR Applied Research Collaboration West, Bristol, England, BS1 2NT, UK

**Keywords:** e-bike, e-scooter, natural experiment, physical activity, e-bike share hire, e-scooter share hire

## Abstract

**Background:**

This study aimed to collect information on e-bike and e-scooter use in areas with and without e-bike (EB) and e-bike plus e-scooter (EB+ES) combined share-hire schemes.

**Methods:**

This study employed a repeated cross-sectional design. An online survey asking questions about demographics, travel, and health was completed by people in August and September 2023 before the schemes were launched in Bristol (EB+ES) and Leeds (EB), with Bradford and Sheffield as control sites. A resurvey was conducted at the same sites one year later, but also in Bath (EB+ES) and Plymouth (EB). We also interviewed eight e-bike and e-scooter users and non-users in Bristol (n=4) and Leeds (n=4).

**Results:**

Following data cleaning, 3771 remained in the baseline sample and 5370 remained in the resurvey sample. The majority of participants reported having never used an e-bike (baseline: 61%; resurvey: 69%) or e-scooter (baseline: 77%; resurvey: 84%). At baseline, the most common e-bike access route was the use of their own e-bike (45%), with access via a share-hire scheme lower at 25%. In the resurvey sample, access levels were similar via a share-hire scheme (38%) and personal e-bikes (36%). The most common e-scooter access route was a share-hire scheme (baseline: 60%; resurvey: 74%). The most common weekly e-bike and e-scooter destinations were leisure/leisure venues, followed by work/education and shopping/errands.

Half said they would not use an e-bike scheme and 63% indicated they would not use an e-scooter scheme. Potential users were willing to walk ~500 m to access an e-bike/e-scooter.

Interviewees generally supported share-hire schemes, seeing them as a good addition to the wider transport offer, but with more support for e-bikes and reservations around e-scooters.

**Conclusions:**

These data will be important for a later evaluation of EB and EB+ES share-hire schemes on public health, social, economic, and environmental factors.

## Plain Language Summary

Physical activity is important for health. Walking or cycling is one way to be physically active. E-bikes are pedal bikes that have battery-powered electric motors. This makes cycling easier while maintaining some physical activity. E-bikes could be an option to increase physical activity. E-scooters run on electric motors, meaning that you do not have to push them. E-scooters may decrease physical activity if they are used instead of walking or cycling.

During the autumn of 2023, Bristol added e-bikes to their e-scooter share-hire scheme. In contrast, Leeds introduced an e-bike only share-hire scheme. Bradford and Sheffield did not use these share-hire schemes. We used a survey to collect information prior to the launch of the schemes. It asked people about themselves and how they travelled between places. One year later, we sent a resurvey to people at the same sites but also to people in Bath and Plymouth. We also interviewed e-bike and e-scooter users and non-users in Bristol and Leeds.

Most had never used an e-bike or e-scooter. Almost half of those who had used an e-bike before said that they used their own e-bike. One year later, a similar number of people said they had used a share-hire scheme (38%) or their own e-bike (36%). Most people who had previously used an e-scooter said it was via a share-hire scheme. People use e-bikes and e-scooters to access leisure, work, education, shopping, and errands. Half said that they would not use an e-bike share-hire scheme and 63% said they would not use an e-scooter share-hire scheme. Interviewees were generally in favour of e-bike and e-scooter share-hire schemes. They showed greater support for e-bikes and had concerns about e-scooters.

We will use the collected data to understand the advantages and disadvantages of these types of schemes in more detail.

## Introduction

The car is still the dominant mode of transport in the UK for all journeys of one mile or more ^1^. However, the harms associated with cars are well established, including ill health and environmental damage ^2^. E-bike and e-scooter hire schemes offer sustainable ways of travel ^3^ and may have health impacts on the user ^4^, but these are unclear ^4– 6^, particularly in the UK context. When referring to e-bikes, we mean cycles that the user must pedal for assistance; throttle-powered electric bicycles were not considered in this paper ^7^. In the UK, the use of e-bikes is legal, but the UK government is yet to make a decision about legalising the use of e-scooters outside trial areas ^8^. The Department for Transport (DfT) launched its e-scooter pilot scheme in 2020, involving 32 UK regions ^9^, with trials recently extended for a fourth time until May 2026 ^8^. Reasons cited for the extension include the need for more evidence on ‘usage, safety and environmental impacts and to explore changing travel patterns since the coronavirus pandemic and as e-scooters become more embedded in public life’ ^8^.

E-bike and e-scooter hire schemes extend travel options, which may in turn affect physical activity ^4, 10, 11^ depending on the transport they replace. Physical activity reduces the risk of chronic diseases ^12^ and all-cause mortality ^13^. The UK Chief Medical Officer recommends that adults engage in 150 minutes of moderate-to-vigorous intensity physical activity (MVPA) per week ^14^. In the UK, 37% of adults do not meet these guidelines ^15^. Increased active travel is associated with a corresponding increase in the overall level of physical activity ^16^. Furthermore, walking and cycling are associated with 11% and 10% reductions in all-cause mortality risk, respectively ^17^.

E-bikes are considered a form of active travel ^18– 20^, which provides at least moderate-intensity physical activity ^18– 20^ and can improve health ^4^. Individuals ride an e-bike for longer and further than a conventional bicycle and therefore experience similar physical activity gains as conventional cyclists ^4, 10^. Improved mental health has also been reported with e-cycling ^21^. There is evidence that e-cycling improves cardiorespiratory fitness ^4^. However, the level of physical activity associated with e-scooter use remains unclear ^22^. E-scootering may affect active travel behaviour through both substitution and complementary effects on active travel ^9, 23^. The precise energy expenditure associated with e-scooter use within share-hire schemes is unclear, with insufficient evidence available for their inclusion in the 2024 compendium of physical activities ^22^. A laboratory-based study (n=42) using an e-scooter mounted on a treadmill found that e-scootering provided significantly less energy expenditure than walking for the same duration ^24^. A small study (n=8) using commercial activity trackers suggested that e-scootering may provide no activity or light activity ^11^. Furthermore, individuals reported disproportionately replacing walking and bike journeys with e-scooters ^11^. 

E-scooter and e-bike share-hire schemes can help connect people with public transport (end-to-end solutions) ^25^. This is important because options are often lacking. Few studies have explored how access to an e-bike or e-scooter affects access to employment, education, or other societal opportunities ^26^. This may offer scope for reducing inequalities, as ethnic minority and low-income users were more likely to report being regular e-scooter users in UK e-scooter trials ^9^. Further, a survey study among share-hire scheme users (n=2402), found that those with protected characteristics or personal challenges were more likely to report benefits to well-being ^27^.

E-bikes and e-scooters are more environmentally friendly than cars ^3, 9^ and peri-urban and rural areas are likely to have the greatest potential for individual carbon savings ^3^. However, charging-related emissions of e-bikes and e-scooters are less environmentally friendly than conventional scootering or cycling ^5^, and share-hire schemes may have greater life-cycle emissions due to fleet management ^28^. Several qualitative studies have found that individuals report using their cars less once they have access to an e-bike ^29^. However, quantitative survey data from the PASTA project exploring e-bike use in nine EU countries found that the primary mode for which e-bikes were substituted depended on the primary mode of transport used in the city at the time ^10^. Chang ^30^ reported that e-scooter trips replaced walking and bicycling trips as often as car trips.

This study aimed to collect information on e-bike and e-scooter use in areas with and without EB and EB+ES combined share-hire schemes. These data will be used in the subsequent evaluation of EB and EB+ES share-hire schemes for public health, social, economic, and environmental factors (NIHR163726; https://www.fundingawards.nihr.ac.uk/award/NIHR163726).

## Methods

### Patient and Public Involvement

The Applied Research Collaboration West (NIHR ARC West) facilitated the recruitment of a combination of e-bike and e-scooter users and non-users for the focus group held during the planning phase of this project. This informed our understanding of what was important to both users and non-users when designing the larger project. We also had a Patient and Public Involvement (PPI) member embedded in the research team who provided important input during research team meetings and advised on documentation, especially that which was publicly facing.

We worked with members from the transport divisions of the local authorities to choose and design the proposed data collection methods for this project. For example, some data collection methods that were originally suggested were deemed likely to be less successful and therefore were not included in the final project. With respect to survey development, local authority members from the Sheffield City Council, Bristol City Council, and Plymouth City Council provided input to improve it. These included adjustments to word choice, restructuring of the landing page, and adjustments to some of the questions.

### Study design and setting

This study employed a repeated cross-sectional design. Although the data include 1316 repeated measures, this study focused on a cross-sectional comparison. The full protocol was registered in the Open Science Framework ( https://osf.io/gq9s8/, DOI 10.17605/OSF.IO/GQ9S8). Reporting follows the STROBE checklist for cross-sectional studies where applicable. We collected baseline data during August and September 2023 prior to the implementation of the two e-bike share-hire schemes. Resurvey data were collected a year later between August and the first week of October 2024. The main project included four major English cities, with baseline and resurvey data. Leeds had no share-hire scheme at baseline, but launched an e-bike share-hire scheme (EB) in mid-September 2023 (baseline data collection was stopped the day before the launch of the scheme). Bristol had an e-scooter share-hire scheme only at baseline but launched a combined e-bike and e-scooter share-hire scheme (EB+ES) in October 2023. Bradford and Sheffield were non-intervention control areas without share-hire schemes. For triangulation purposes, we added two additional cities to the resurvey phase. These were Plymouth, which launched an EB scheme in late March 2023, and Bath, which had an e-scooter only share-hire scheme but extended it to an EB+ES scheme in late September 2023.

### Survey data collection

***Online survey.*** The baseline survey and resurvey, developed in consultation with council communication and transport teams from the four local authorities, contained 32 questions for the baseline survey and 41 questions for the resurvey. The full questionnaire information can be found in the protocol ( https://osf.io/gq9s8/, DOI 10.17605/OSF.IO/GQ9S8). They inquired about demographics (age, gender, ethnicity, employment, income, and postcode), physical activity volume (self-reported minutes of moderate-to-vigorous physical activity (MVPA)), use of e-bikes or e-scooters, active travel and other travel, distance travelled by different transport modes, substitution of travel modes, quality of life, and access to venues and services. Written informed consent was obtained from all participants, as outlined in the ethics and consent sections below.

***Baseline Survey Data Collection.*** Baseline recruitment targeted residents aged 16 years and over of the two intervention local authorities, Leeds and Bristol, and the two control local authorities, Sheffield and Bradford. The intervention and control sites were similar with respect to the proportion reporting very good health (50%, 51%, 47%, 46%) and income deprivation (14%, 14%, 16%, 19%) ^31^. 

The primary mode of distribution for our baseline survey was through advertisements with a link to our survey distributed via council-administered newsletters and/or mailing lists and council-administered social media. We supplemented this with secondary methods. First, paid-for Facebook advertising, which has extensive reach with 79% of UK adults who go online using Facebook and 45% using it on a daily basis ^32^. Second, relevant local stakeholder groups and gatekeepers of relevant social media groups in each study area facilitated distribution to their members to improve reach to seldom heard voices. Third, we used a prize draw in each region as an incentive, as including entry into prize draws has been shown to improve response rates to surveys ^33^.

***Resurvey survey data collection.*** During the resurvey, we included the original sites and extended them to collect resurvey data in two further cities: Plymouth (EB) and Bath (EB+ES) to triangulate data with Leeds (EB) and Bristol (EB+ES), respectively. For the resurvey, we employed the same survey data collection methods used at baseline (as outlined above). However, we found that engagement with the Bradford population at baseline was considerably more challenging than that at other sites, as evidenced by the lower survey response rate. Given that the Bradford site contains much higher ethnic diversity, it is important to increase its representation to ensure diversity and inclusion and allow a sufficient sample size to explore inequalities. Therefore, we partnered with CNET Bradford ( https://cnet.org.uk/), a charity and company that supports the voluntary, community, and social enterprise sectors in Bradford, to engage the local community and improve response rates at resurvey. Additional channels included information events in communities with lower response rates at baseline, facilitated by trusted community advocates, and trusted advocates attending places the target population visited (e.g., supermarkets, libraries, youth groups) to promote the survey and offer support in completing the survey if required. As Bath had a much smaller population size than the other sites, we also supplied physical posters containing a QR code link to the online survey in public places to increase survey reach.

***Sample size.*** For the purposes of the later full evaluation (NIHR163726; https://www.fundingawards.nihr.ac.uk/award/NIHR163726), we estimated the sample size required to detect a difference in self-reported MVPA between the evaluation arms with and without EB schemes of between 5% and 15%, based on 80% power and 5% significance level. These represent differences of approximately 15–50 min in total weekly MVPA, which are achievable and in line with published research for achieving health benefits ^34^. For example, a difference of 30 min is equivalent to an extra 10 min of MVPA three times a week. Originally, we used published data (M (mean) = 258 min, standard deviation (SD) = 214 min) to estimate a sample size of 1080 per arm required to detect a 10% increase in weekly MVPA (26 min), assuming normally distributed data. Using the observed baseline data collected in August and September 2023, we produced revised estimates (M = 305 min, SD = 257 min per week) for an analysis based on a lognormal distribution, which better reflects the highly skewed nature of the data. Sample sizes of 1400–2400 in each arm were calculated to be sufficient to detect a difference of 25–30 min of MVPA.

***Survey data cleaning.*** To ensure the validity of the survey respondents residing in one of the six study sites (Bristol, Leeds, Bradford, Sheffield, Bath, or Plymouth) and to remove potential contamination effects, for example, Leeds respondents accessing the Bradford survey (or vice versa), participants were assigned a site based on the local authority of their home postcode. Respondents with postcodes outside the local authority areas were removed. Respondents who provided no postcode but reported that they 'lived in the city' remained allocated to the site based on the completed site survey.

Duplicate entries were classified as respondents who provided identical names, email addresses, or telephone numbers. All entries after the first (based on the time of survey completion) were removed if the number of duplicates exceeded two. For only two duplicate entries, data available in entry two but not in entry one were added to entry one, and the second entry was then removed.

Implausible demographic data values (considered for age>110 years, weight <30 kg or >400 kg and height <1.2m or >2.2m ^35^) were set to missing ^36^. Physical activity levels were grouped according to meeting (≥ 150 minutes per week) or not meeting (<150 minutes per week) the UK Chief Medical Officer Physical Activity Guidelines ^14^. Respondents were assigned to the 2019 English Index of Multiple Deprivation (IMD) quintiles based on their home postcode ^37^. Finally, missing demographic data at resurvey for those with repeated measures were replaced with baseline data, with age increased by one year.

***Survey data analysis.*** Categorical data are presented as n (%) and continuous data as mean (SD) if approximately normally distributed and median (IQR: Q1, Q3) otherwise. Data are presented for both the baseline and resurvey, where applicable. Basic descriptive analyses were performed to gain an understanding of typical e-bike and e-scooter use by each local authority. Sample demographic characteristics are described for all respondents at each site and overall. Demographics included: gender (male, female), age (16–34, 35–44, 45–54, 55–64, 65+), ethnicity (White, Black, Asian, Mixed, Other), employment (working (employed or self-employed part- or full-time), not working), education (degree level or above, below degree level), household income (<£7000, £7000–£19,999, £20,000–£29,999, £30,000–£39,999, £40,000–£59,999, £60,000+), IMD quintiles, household type (single, couple, family, other), health conditions (yes, no), physical activity level (meeting guidelines, not meeting guidelines), BMI (<25, 25–29.9, 30+ kg/m ^2^), ever e-bike use (yes, no), and ever e-scooter use (yes, no). Sample demographic characteristics including gender, age, and health conditions were also presented for those reporting ever having used an e-bike/e-scooter previously (‘ever users’). It is important to note that the purpose of the data collection was not to produce representative estimates of the demographics of e-bike or e-scooter users, but to allow a specific evaluation of these schemes, which will be analysed in detail in a subsequent NIHR project (NIHR163726; https://www.fundingawards.nihr.ac.uk/award/NIHR163726). However, to explore how representative the sample is of the wider population, sample demographic characteristics were compared descriptively against census 2021 data ^31^ for age, gender, ethnicity, and highest education level for each site and for all sites combined.

We asked respondents to provide information on transport use during a typical week and presented the frequency (five or more times per week, 3–4 times per week, 1–2 times per week, less than once per week, never) of type of transport use (car, walking, bus, train, conventional bicycle, e-bike, e-scooter) for each site and overall. Of the sites we considered, e-scooter use is only legal in Bath and Bristol; we have only reported the frequency of e-scooter use data for Bristol due to the very low numbers in other sites.

We asked the e-bike and e-scooter users how they had accessed them: through a share scheme, loan scheme, hire on a day-to-day basis, or a personal e-bike or e-scooter. We also asked them to indicate which venues they accessed on a weekly basis using an e-bike or e-scooter (work/education, healthcare appointment, public transport, shopping/errands, visiting friends, leisure/leisure venues, and job interviews/job centres). Finally, we asked participants to report the distance they would be willing to walk or wheel to access a share-hire scheme e-bike or e-scooter. Frequencies were reported according to the following categories: I would not use a share scheme, less than 100 meters (about 1 min), 100 to 250 meters (about 2.5 minutes), 250 to 500 meters (about 4 minutes), 500 meters to 1 km (about 7 minutes), more than 1 km (more than 10 minutes), and ‘don’t know’.

### Interviews

***Data collection.*** Respondents to the baseline data collection survey were invited to express their interest in a qualitative interview to discuss their views on the introduction of EB and EB+ES share-hire schemes and their experience. Four respondents living in Leeds and four living in Bristol were selected to provide a set with a mix of characteristics based on age, ethnicity, gender, disability, and whether they were users or non-users of the schemes. Interviews of approximately one hour in duration were conducted online (n=3) or in person (n=5) between three and four months after the introduction of the share-hire schemes. The interviews were semi-structured and investigated household travel behaviour, awareness of and attitudes to e-bike/e-scooter schemes in the local context, and prospects for travel behaviour change in the following 12 months (incorporating e-bike and/or e-scooter use and under what conditions). All interviews were audio-recorded. The opportunity was also taken to ask participants about their experience of completing the baseline survey to enable researchers to consider any adjustments that may be required. Informed consent was obtained from all participants as outlined below. 

***Data analysis.*** Audio recordings of the interviews were transcribed using Otter.ai and then checked for accuracy by the researcher responsible for conducting the interviews. These were sent to participants, who were asked to confirm if there were any inaccuracies within ten days of receiving them. The researcher then wrote a series of vignettes that involved compiling biographical summaries and accounts of the topics covered during semi-structured interviews, illustrated with verbatim quotes. These were ‘sense-checked’ by a coresearcher who read the researcher vignettes in tandem with the source transcripts.

## Ethical approval

Ethical approval for this study was granted by the School for Policy Studies Research Ethics Committee of the University of Bristol (SPSREC/2223/362) on 28 ^th^ July 2023. The study officially started on 1 ^st^ August 2023. Permission to collect the resurvey data was granted as an ethics amendment to the SPSREC/2223/362 on 29 ^th^ May 2024. Ethical approval also included approval for a prize draw for each region as an incentive to increase participation at both the baseline and resurvey. These prize draws were included following discussions with local authorities.

## Consent

All study participants provided their consent prior to taking part in the study. Survey participants were required to provide typed electronic consent online. First, they were presented with an information sheet informing them about the project and were provided with an email address to contact if they had any queries. They were then presented with a list of consent statements. If they agreed with these statements, they were asked to indicate this electronically and were then allowed to proceed to the online survey. The interview participants signed either an electronic or paper consent form prior to the start of the interview. They were also asked to verbally repeat their consent at the start of the interview.

## Results

### Survey data

**Figure 1.**
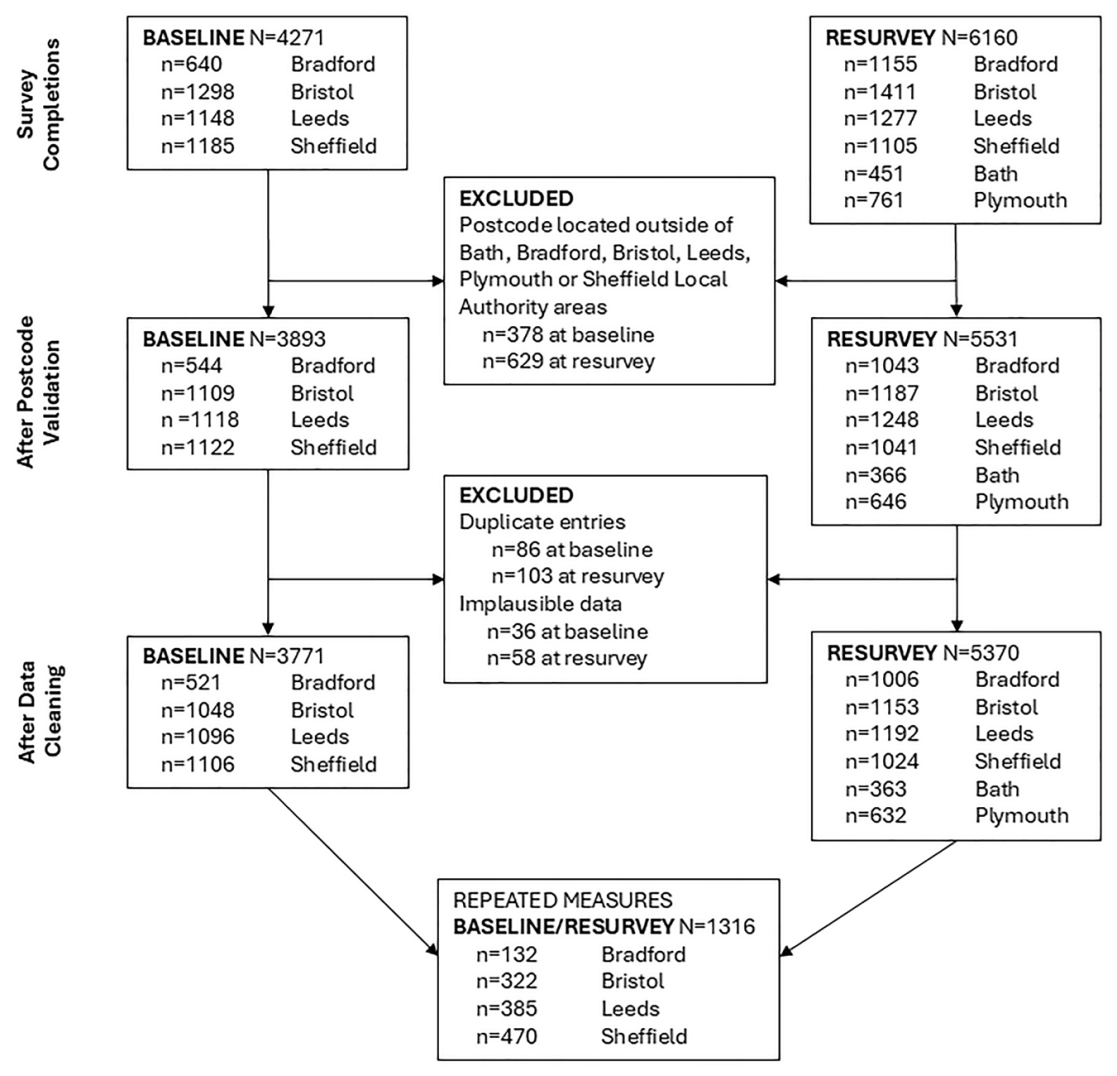
Study flowchart.

At baseline, 4271 online surveys were completed. Following data cleaning and duplicate removal, N=3771 (n=1048 for Bristol, n=1096 for Leeds, n=521 for Bradford, and n=1106 for Sheffield) remained for the baseline data analysis. In the resurvey, 6160 online surveys were completed. After data cleaning and duplicate removal, N=5370 (n=1153 for Bristol, n=1192 for Leeds, n=1006 for Bradford, n=1024 for Sheffield, n=363 for Bath, and n=632 for Plymouth) remained for the resurvey data analysis. Figure 1 illustrates the flow of participants from recruitment to data analysis.

***Descriptive characteristics.*** Generally, there were slightly more women than men in both the baseline (~52% vs. ~46%) and resurvey (60% vs. 38%) samples (Extended Data Tables 1 and 2). At baseline, older age groups (>55 years) were more frequent in Leeds and Sheffield, and younger age groups were more common in Bristol and Bradford (<45 years) (Extended Data Table 1). At resurvey, older people were more frequent in Leeds, Bradford, and Sheffield, with younger people in Bristol and Bath (16–34) (Extended Data Table 2). The sample was predominantly of white ethnicity (92%, baseline; 90% resurvey overall) (Extended Data Tables 1 and 2). Most sites reported 91-95% white ethnicity but this was lower in Bradford respondents (baseline: 83%; resurvey: 76%), who reported 11% Asian ethnicity at baseline and 17% Asian ethnicity at resurvey (Extended Data Tables 1 and 2).

The majority of both the baseline and resurvey samples were in work (baseline: 66%; resurvey: 64%) (Extended Data Tables 1 and 2). The sample was skewed towards higher education, with 71% at baseline (75% at resurvey) educated to degree level or above (Extended Data Tables 1 and 2). The sample was also skewed towards higher household income categories at both the baseline and resurvey (>£40k) (Extended Data Tables 1 and 2). IMD showed a fairly even spread overall; however, deprivation levels were high in Bradford and Plymouth, with lower deprivation levels in Bath and Sheffield (Extended Data Tables 1 and 2).

Across all sites, people predominantly reported living as a couple (baseline: 38%; resurvey: 40%) or as a family with children under 18 years of age (baseline: 28%; resurvey: 23%) (Extended Data Tables 1 and 2). Approximately two-thirds of the respondents reported no health conditions (baseline: 67%; resurvey: 63%) (Extended Data Tables 1 and 2). Slightly more participants (73%) reported meeting the physical activity guidelines in the baseline sample than in the resurvey sample (68%; Extended Data Tables 1 and 2). Approximately one-fifth of the overall sample reported living with obesity (baseline: 22%; resurvey: 21%), and approximately one-third reported living with overweight (baseline: 31%; resurvey: 33%) (Extended Data Tables 1 and 2).

The majority of the sample reported having never used an e-bike, ranging from 50% in Bradford to 71% in Leeds at baseline (61% overall), and 64% in Sheffield/Plymouth to 79% in Bradford at resurvey (69% overall). Similar percentages had never used an e-scooter (baseline 77%: 55% in Bristol, 89% in Sheffield; resurvey: 84%: 70% in Bristol, 93% in Bradford) (Extended Data Tables 1 and 2). Among those who had ever used an e-bike, 53% were men and 45% were women (baseline), with similar proportions of ever e-bike users in the resurvey sample (48% vs. 50%, respectively) ( Table 1 and Table 2). Ever e-bike users comprised more 16–35-year-olds (baseline: 32%; resurvey: 28%) and fewer aged 65+ (baseline:12%; resurvey:14%; Table 1 and Table 2). Among those who had ever used an e-bike, 28% reported living with health conditions in the baseline sample ( Table 1), with similar percentages in the resurvey sample (30%; Table 2).

**Table 1.** Respondent characteristics of ever users of e-bikes at baseline.

	Bristol (N=488)	Leeds (N=316)	Bradford (N=261)	Sheffield (N=384)	All (N=1449)
	n (%)	n (%)	n (%)	n (%)	n (%)
Gender *					
Female	228 (48%)	129 (42%)	108 (43%)	178 (47%)	643 (45%)
Male	251 (52%)	174 (56%)	131 (52%)	190 (50%)	746 (53%)
**Age**					
16–34	238 (50%)	66 (22%)	96 (38%)	56 (15%)	456 (32%)
35–44	106 (22%)	58 (19%)	67 (26%)	80 (21%)	311 (22%)
45–54	52 (11%)	67 (22%)	36 (14%)	78 (21%)	233 (17%)
55–64	38 (8%)	63 (21%)	30 (12%)	113 (30%)	244 (17%)
65+	39 (8%)	51 (17%)	24 (9%)	49 (13%)	163 (12%)
**Health conditions**					
Yes	81 (18%)	86 (29%)	39 (18%)	121 (33%)	321 (28%)
No	359 (82%)	208 (71%)	175 (82%)	247 (67%)	828 (72%)

* percentages do not sum to 100 due to small number of reported 'other' genders Cell counts<10 removed to preserve data confidentiality Numbers may not equal 100% due to rounding

**Table 2.** Respondent characteristics of ever users of e-bikes at resurvey.

	Bristol (N=362)	Leeds (N=346)	Bradford (N=212)	Sheffield (N=371)	Bath (N=119)	Plymouth (N=230)	All (N=1640)
	n (%)	n (%)	n (%)	n (%)	n (%)	n (%)	n (%)
Gender *							
Female	182 (51%)	161 (47%)	122 (58%)	184 (50%)	64 (55%)	101 (44%)	814 (50%)
Male	166 (46%)	173 (51%)	85 (40%)	173 (47%)	51 (44%)	126 (55%)	774 (48%)
**Age**							
16–34	122 (34%)	82 (24%)	46 (22%)	76 (21%)	48 (40%)	80 (35%)	454 (28%)
35–44	77 (21%)	60 (17%)	34 (16%)	82 (22%)	16 (13%)	34 (15%)	303 (19%)
45–54	57 (16%)	69 (20%)	45 (21%)	72 (20%)	19 (16%)	41 (18%)	303 (19%)
55–64	52 (14%)	76 (22%)	49 (23%)	97 (26%)	18 (15%)	48 (21%)	340 (21%)
65+	54 (15%)	58 (17%)	37 (18%)	41 (11%)	18 (15%)	27 (12%)	235 (14%)
**Health conditions**							
Yes	91 (27%)	95 (30%)	59 (31%)	125 (36%)	29 (25%)	62 (29%)	461 (30%)
No	244 (73%)	221 (70%)	132 (69%)	227 (64%)	85 (75%)	150 (71%)	1059 (70%)

* percentages do not sum to 100 due to small number of reported 'other' genders Cell counts<10 removed to preserve data confidentiality Numbers may not equal 100% due to rounding

Among those who had ever used an e-scooter, 54% were men and 43% were women in the baseline sample. The proportion of ever e-scooter users in the resurvey sample was slightly higher in women (51%) than in men (46%) overall ( Table 3 and Table 4). There was a higher proportion of young ever e-scooter users and a lower proportion of old ever e-scooter users in both the baseline and resurvey samples (48% were aged 16–35 years vs 3% aged 65+ years at baseline, and 48% were aged 16–35 years vs 6% aged 65+ years at resurvey) ( Table 3 and Table 4). Among the ever users of e-scooters, 22% reported having a health condition in the baseline sample ( Table 3) and 25% reported having a health condition in the resurvey sample ( Table 4).

**Table 3.** Respondent characteristics of ever users of e-scooters at baseline.

	Bristol (N=471)	Leeds (N=141)	Bradford (N=146)	Sheffield (N=116)	All (N=874)
	n (%)	n (%)	n (%)	n (%)	n (%)
Gender *					
Female	212 (46%)	56 (41%)	44 (32%)	52 (46%)	364 (43%)
Male	247 (53%)	79 (58%)	82 (59%)	56 (50%)	464 (54%)
**Age**					
16–34	234 (51%)	59 (43%)	80 (56%)	40 (35%)	413 (48%)
35–44	121 (26%)	35 (25%)	40 (28%)	26 (23%)	222 (26%)
45–54	60 (13%)	18 (13%)	14 (10%)	23 (20%)	115 (13%)
55–64	32 (7%)	18 (13%)	-	19 (17%)	76 (9%)
65+	12 (3%)	-	-	-	27 (3%)
**Health conditions**					
Yes	76 (18%)	33 (26%)	16 (15%)	41 (38%)	166 (22%)
No	344 (82%)	96 (74%)	90 (85%)	67 (62%)	597 (78%)

* percentages do not sum to 100 due to small number of reported 'other' genders Cell counts<10 removed to preserve data confidentiality Numbers may not equal 100% due to rounding

**Table 4.** Respondent Characteristics of ever users of e-scooters at resurvey.

	Bristol (N=338)	Leeds (N=137)	Bradford (N=72)	Sheffield (N=132)	Bath (N=84)	Plymouth (N=76)	All (N=839)
	n (%)	n (%)	n (%)	n (%)	n (%)	n (%)	n (%)
**Gender** *							
Female	172 (51%)	67 (49%)	35 (49%)	69 (52%)	47 (56%)	35 (46%)	425 (51%)
Male	154 (46%)	66 (49%)	36 (50%)	55 (42%)	33 (39%)	40 (53%)	384 (46%)
**Age**							
16–34	164 (49%)	59 (43%)	38 (53%)	58 (44%)	42 (50%)	42 (55%)	403 (48%)
35–44	71 (21%)	27 (20%)	11 (15%)	24 (18%)	12 (14%)	-	154 (18%)
45–54	49 (14%)	25 (18%)	11 (15%)	26 (20%)	18 (21%)	12 (16%)	141 (17%)
55–64	35 (10%)	15 (11%)	-	15 (11%)	10 (12%)	-	92 (11%)
65+	19 (6%)	10 (7%)	-	-	-	-	48 (6%)
**Health conditions**							
Yes	82 (26%)	27 (21%)	17 (26%)	48 (37%)	15 (19%)	-	197 (25%)
No	235 (74%)	99 (79%)	49 (74%)	81 (63%)	64 (81%)	48 (86%)	576 (75%)

* percentages do not sum to 100 due to small number of reported 'other' genders Cell counts <10 removed to preserve data confidentiality Numbers may not equal 100% due to rounding

There were more women in both the baseline (53%) and resurvey (61%) samples compared to census 2021 data (52%). Our sample had fewer young people (16-34 years) overall (baseline: 21%; resurvey: 20% in the sample vs. 35% in census 2021 data). There was a higher proportion of those with white ethnicity in our baseline (92%) and resurvey (90%) samples compared to census 2021 data (81%). The sample was skewed towards higher education, with 71% of the baseline sample and 75% of the resurvey sample reporting being educated to degree level or above versus the expected 34% in the census 2021 data (Extended Data Table 3).

***Typical transport use.*** In all the cities combined, the most common transport modes typically used each week were cars and walking. At baseline, car use of five or more times per week was reported most frequently in Bradford (41%) and Leeds (40%), with lower proportions in Sheffield (31%) and Bristol (22%) ( Table 5). Trends were similar in the resurvey: Bradford (48%), Leeds (37%), Plymouth (32%), Sheffield (27%), Bath (21%), and Bristol (17%) (Extended Data Table 4). In the baseline sample, the proportion of participants reporting walking on five or more days per week ranged from 67% in Sheffield to 59% in Bradford (64% overall) ( Table 5). In the resurvey sample, 79% of the Bath sample reported walking five or more days per week down to 57% in Bradford (67% overall) (Extended Data Table 4).

**Table 5.** Respondent transport use in a typical week at baseline.

		Bristol (N=1048)	Leeds (N=1096)	Bradford (N=521)	Sheffield (N=1106)	All (N=3771)
Transport mode	Frequency	n (%)	n (%)	n (%)	n (%)	n (%)
Car	5+ times per week	225 (22%)	428 (40%)	204 (41%)	328 (31%)	1185 (33%)
	3–4 times per week	181 (18%)	254 (24%)	81 (16%)	222 (21%)	738 (20%)
	1–2 times per week	290 (29%)	177 (17%)	97 (20%)	238 (22%)	802 (22%)
	Less than once per week	219 (22%)	140 (13%)	70 (14%)	193 (18%)	622 (17%)
	Never	103 (10%)	60 (6%)	42 (9%)	84 (8%)	289 (8%)
Walking	5+ times per week	628 (63%)	656 (64%)	280 (59%)	698 (67%)	2262 (64%)
	3–4 times per week	155 (16%)	185 (18%)	85 (18%)	166 (16%)	591 (17%)
	1–2 times per week	108 (11%)	113 (11%)	61 (13%)	108 (10%)	390 (11%)
	Less than once per week	76 (8%)	45 (4%)	15 (3%)	28 (3%)	164 (5%)
	Never	31 (3%)	25 (2%)	31 (7%)	41 (4%)	128 (4%)
Bus	5+ times per week	73 (8%)	94 (10%)	35 (8%)	73 (8%)	275 (8%)
	3–4 times per week	103 (11%)	85 (9%)	38 (9%)	94 (10%)	320 (10%)
	1–2 times per week	206 (22%)	151 (16%)	67 (16%)	182 (19%)	606 (19%)
	Less than once per week	391 (41%)	419 (44%)	148 (35%)	383 (40%)	1341 (41%)
	Never	174 (18%)	206 (22%)	139 (33%)	219 (23%)	738 (23%)
Train	5+ times per week	22 (2%)	-	12 (3%)	-	47 (2%)
	3–4 times per week	34 (4%)	15 (2%)	19 (5%)	11 (1%)	79 (3%)
	1–2 times per week	112 (12%)	80 (9%)	48 (11%)	46 (5%)	286 (9%)
	Less than once per week	452 (50%)	497 (56%)	198 (47%)	532 (60%)	1679 (54%)
	Never	285 (32%)	289 (33%)	148 (35%)	292 (33%)	1014 (33%)
Conventional bicycle	5+ times per week	115 (13%)	43 (5%)	33 (8%)	97 (11%)	288 (9%)
	3–4 times per week	135 (15%)	56 (6%)	33 (8%)	77 (9%)	301 (10%)
	1–2 times per week	163 (18%)	87 (10%)	66 (16%)	85 (10%)	401 (13%)
	Less than once per week	109 (12%)	161 (18%)	70 (17%)	133 (15%)	473 (15%)
	Never	397 (43%)	534 (61%)	205 (50%)	503 (56%)	1639 (53%)
E-bike	5+ times per week	82 (9%)	49 (6%)	27 (7%)	65 (7%)	223 (7%)
	3–4 times per week	93 (10%)	26 (3%)	50 (12%)	53 (6%)	222 (7%)
	1–2 times per week	99 (11%)	47 (5%)	46 (11%)	43 (5%)	235 (8%)
	Less than once per week	63 (7%)	43 (5%)	35 (9%)	51 (6%)	192 (6%)
	Never	566 (63%)	703 (81%)	255 (62%)	669 (76%)	2193 (72%)
E-scooter	5+ times per week	70 (8%)	-	-	-	-
	3–4 times per week	69 (8%)	-	-	-	-
	1–2 times per week	110 (12%)	-	-	-	-
	Less than once per week	157 (17%)	-	-	-	-
	Never	500 (55%)	-	-	-	-

Cell counts <10 removed to preserve data confidentiality Numbers may not equal 100% due to rounding

The majority of respondents reported never using a conventional bicycle in both the baseline (53%) and resurvey (67%) samples ( Table 5 and Extended Data Table 4). E-bike use was less common than conventional bicycle use, with 72% of the baseline sample and 85% of the resurvey sample reporting never using one in a typical week.

Overall, 8% reported using e-bikes 1–2 times per week and 7% reported using them 3–4 times per week in the baseline sample, with the corresponding proportions in the resurvey sample being 3%. In the baseline sample, lower proportions of e-bike use in a typical week were reported in Leeds (19%) and Sheffield (24%), with higher proportions reported in Bradford (38%) and Bristol (37%) ( Table 5). In the resurvey sample, proportions were lower and similar across all cities, with around 15% overall reporting ever using an e-bike in a typical week (Extended Data Table 4).

Regular e-scooter use in Bristol was uncommon, with 55% at baseline and 85% in the resurvey sample reporting never using one in a typical week. When respondents reported using an e-scooter, they most commonly reported using one less than once per week (17% baseline and 10% resurvey) ( Table 5 and Extended Data Table 4).

***E-bike access and destinations.*** At baseline, the most common e-bike access route was the use of one’s own e-bike (45% overall; Bristol 37%; Leeds 49%; Bradford 48%; Sheffield 51%). Prior access via a share-hire scheme was 25% overall at baseline, with higher proportions reporting this route in Bristol (38%) and Bradford (32%), with respondents from Leeds (17%) and Sheffield (11%) indicating lower proportions ( Table 6). In the resurvey, the overall proportions reporting access via a share-hire scheme (38%) or their own personal e-bike (36%) were similar ( Table 7). However, at the individual city level, Leeds was the only site to report similar levels of access via a share-hire scheme (38%) or their own personal e-bike (39%). Bradford (41%) and Sheffield (40%) still indicated high access via their own personal e-bike at resurvey, whereas for respondents from Bristol (49%), Bath (42%), and Plymouth (65%), the most commonly reported access route was via a share-hire scheme. Note that this refers to access at any point in time over their lifetime, and the access routes are not mutually exclusive.

**Table 6.** E-bike access routes and destinations in respondents who reported having ever used an e-bike (baseline).

		Bristol	Leeds	Bradford	Sheffield	All
		n (%)	n (%)	n (%)	n (%)	n (%)
Access route	Share scheme (for example Beryl, Voi)	179 (38%)	54 (17%)	78 (32%)	42 (11%)	353 (25%)
	Loan scheme (for example loaned for a period of a few weeks)	78 (16%)	20 (6%)	20 (8%)	70 (19%)	188 (13%)
	Hired on a day-to-day basis	90 (19%)	58 (19%)	53 (21%)	58 (15%)	259 (18%)
	Personal e-bike (own)	177 (37%)	151 (49%)	118 (48%)	194 (51%)	640 (45%)
	Personal e-bike (borrowed)	68 (14%)	83 (27%)	59 (24%)	103 (27%)	313 (22%)
	Total	476	309	247	377	1409
Weekly destination	Work/education	271 (57%)	94 (30%)	98 (40%)	148 (39%)	611 (43%)
	Healthcare appointment	89 (19%)	34 (11%)	44 (18%)	66 (17%)	233 (16%)
	Public transport	79 (17%)	15 (5%)	37 (15%)	29 (8%)	160 (11%)
	Shopping/errands	195 (41%)	110 (35%)	99 (40%)	182 (48%)	586 (41%)
	Visiting friends	110 (23%)	47 (15%)	72 (29%)	91 (24%)	320 (23%)
	Leisure/leisure venue	250 (52%)	125 (40%)	123 (50%)	206 (54%)	704 (50%)
	Job interviews/job centre	98 (21%)	14 (5%)	41 (17%)	-	158 (11%)
	None of these	142 (29%)	172 (52%)	82 (32%)	183 (46%)	579 (39%)
	Total	477	310	247	379	1413

Cell counts <10 removed to preserve data confidentiality Numbers may not equal 100% due to rounding

**Table 7.** E-bike access routes and destinations in respondents who reported having ever used an e-bike (resurvey).

		Bristol	Leeds	Bradford	Sheffield	Bath	Plymouth	All
		n (%)	n (%)	n (%)	n (%)	n (%)	n (%)	n (%)
Access route	Share scheme (for example Beryl, Voi)	175 (49%)	129 (38%)	43 (21%)	76 (21%)	50 (42%)	147 (65%)	620 (38%)
	Loan scheme (for example loaned for a period of a few weeks)	25 (7%)	21 (6%)	12 (6%)	77 (21%)	13 (11%)	-	152 (9%)
	Hired on a day-to-day basis	62 (17%)	71 (21%)	46 (22%)	81 (22%)	21 (18%)	20 (9%)	301 (19%)
	Personal e-bike (own)	110 (31%)	133 (39%)	86 (41%)	145 (40%)	39 (33%)	64 (28%)	577 (36%)
	Personal e-bike (borrowed)	83 (23%)	89 (26%)	70 (34%)	107 (29%)	36 (30%)	48 (21%)	433 (27%)
	Total	359	344	208	364	119	227	1621
Weekly destination	Work/education	89 (25%)	100 (29%)	47 (22%)	96 (26%)	36 (30%)	79 (35%)	447 (27%)
	Healthcare appointment	42 (12%)	39 (11%)	20 (9%)	42 (11%)	25 (21%)	29 (13%)	197 (12%)
	Public transport	24 (7%)	23 (7%)	10 (5%)	13 (4%)	-	25 (11%)	98 (6%)
	Shopping/errands	97 (27%)	101 (29%)	54 (25%)	124 (34%)	48 (40%)	84 (37%)	508 (31%)
	Visiting friends	62 (17%)	46 (13%)	38 (18%)	55 (15%)	24 (20%)	33 (14%)	258 (16%)
	Leisure/leisure venue	139 (39%)	123 (36%)	72 (34%)	127 (34%)	44 (37%)	98 (43%)	603 (37%)
	Job interviews/job centre	-	-	-	-	-	-	25 (2%)
	None of these	196 (54%)	189 (54%)	119 (55%)	210 (56%)	70 (57%)	97 (42%)	881 (53%)
	Total	361	344	212	370	119	228	1634

Cell counts <10 removed to preserve data confidentiality Numbers do not equal 100% due to being able to choose multiple options

Overall, the most common weekly e-bike destination was leisure/leisure venue at both baseline (50%) and resurvey (37%), followed by work/education (baseline: 43%; resurvey: 27%), and shopping/errands (baseline: 41%; resurvey: 31%) ( Table 6 and Table 7). Note that the destinations are not mutually exclusive.

***E-scooter access and destinations.*** The most common e-scooter access route was via a share-hire scheme (e.g., Beryl/Voi/Tier/Dott), with 60% overall at baseline and 74% overall at resurvey (slightly higher in Bristol) ( Table 8 and Table 9). However, this differed for Bradford, where the most common e-scooter access route was the use of either their own or a borrowed personal e-scooter (baseline: 51%; resurvey: 47%), followed by a share-hire scheme (baseline: 33%; resurvey: 32%) ( Table 8 and Table 9). In Bristol, the most common weekly e-scooter destination was for leisure/a leisure venue (baseline: 61%; resurvey: 30%), followed by work/education (baseline: 48%; resurvey: 18%), and shopping/errands (baseline: 46%; resurvey: 18%) ( Table 8 and Table 9).

**Table 8.** E-scooter access routes and destinations in respondents who reported having ever used an e-scooter (baseline).

		Bristol	Leeds	Bradford	Sheffield	All
		n (%)	n (%)	n (%)	n (%)	n (%)
Access route	Share scheme (for example Beryl, Voi)	329 (70%)	73 (54%)	45 (33%)	65 (58%)	512 (60%)
	Loan scheme (for example loaned for a period of a few weeks)	60 (13%)	-	23 (17%)	-	87 (10%)
	Hired on a day-to-day basis	86 (18%	40 (29%)	33 (24%)	27 (24%)	186 (22%)
	Personal e-scooter (own/borrowed)	90 (19%)	33 (24%)	72 (51%)	27 (23%)	222 (26%)
	Total	467	136	136	113	852
Weekly destination	Work/education	224 (48%)	-	-	-	-
	Healthcare appointment	84 (18%)	-	-	-	-
	Public transport	95 (20%)	-	-	-	-
	Shopping/errands	218 (46%)	-	-	-	-
	Visiting friends	131 (28%)	-	-	-	-
	Leisure/leisure venue	285 (61%)	-	-	-	-
	Job interviews/job centre	51 (11%)	-	-	-	-
	None of these	96 (20%)	-	-	-	-
	Total	469	-	-	-	-

We have only reported the weekly destinations for Bristol as this is the only city with an e-scooter scheme Cell counts <10 removed to preserve data confidentiality Numbers do not equal 100% due to being able to choose multiple options

**Table 9.** E-scooter access routes and destinations in respondents who reported having ever used an e-scooter (resurvey).

		Bristol	Leeds	Bradford	Sheffield	Bath	Plymouth	All
		n (%)	n (%)	n (%)	n (%)	n (%)	n (%)	n (%)
Access route	Share scheme (for example Beryl, Voi)	295 (88%)	92 (68%)	23 (32%)	83 (64%)	72 (86%)	54 (73%)	619 (74%)
	Loan scheme (for example loaned for a period of a few weeks)	-	-	-	-	-	-	15 (2%)
	Hired on a day-to-day basis	43 (13%)	14 (10%)	17 (24%)	30 (23%)	-	-	116 (14%)
	Personal e-scooter (own/ borrowed)	22 (7%)	38 (28%)	34 (47%)	23 (17%)	13 (15%)	20 (26%)	150 (18%)
	Total	337	136	71	130	84	74	832
Weekly destination	Work/education	60 (18%)	-	-	-	-	-	-
	Healthcare appointment	10 (3%)	-	-	-	-	-	-
	Public transport	33 (10%)	-	-	-	-	-	-
	Shopping/errands	59 (18%)	-	-	-	-	-	-
	Visiting friends	41 (12%)	-	-	-	-	-	-
	Leisure/leisure venue	102 (30%)	-	-	-	-	-	-
	Job interviews/job centre	-	-	-	-	-	-	-
	None of these	185 (55%)	-	-	-	-	-	-
	Total	337	-	-	-	-	-	-

We have only reported the weekly destinations for Bristol as this is the only city with an e-scooter scheme Cell counts <10 removed to preserve data confidentiality Numbers do not equal 100% due to being able to choose multiple options

***Share scheme access distances.*** Half of the respondents indicated that they would not use an e-bike share-hire scheme, and 63% indicated that they would not use an e-scooter share-hire scheme. Of those who indicated that they would potentially use a share-hire scheme, 34% were willing to walk/wheel up to 500 m (32% for an e-scooter) and 28% were willing to walk/wheel up to 1 km to access an e-bike (25% for an e-scooter). Very few respondents were willing to walk/wheel more than 1 km to access an e-bike (14%) or e-scooter (12%) via a share-hire scheme ( Table 10).

**Table 10.** Distances participants in overall sample report being willing to walk (or wheel) to access an e-bike or e-scooter share scheme.

	E-bike (all)	E-bike (potential users)	E-scooter (all)	E-scooter (potential users)
Distance	n (%)	n (%)	n (%)	n (%)
I would not use a share scheme	2653 (50%)	-	3343 (63%)	-
Less than 100 metres (about 1 minute)	138 (3%)	138 (6%)	139 (3%)	139 (8%)
100 to 250 metres (about 2.5 minutes)	443 (8%)	443 (18%)	389 (7%)	389 (23%)
250 to 500 metres (about 4 minutes)	822 (15%)	822 (34%)	546 (10%)	546 (32%)
500 metres to 1 kilometre (about 7 minutes)	676 (13%)	676 (28%)	437 (8%)	437 (25%)
More than 1 kilometre (more than 10 minutes)	352 (7%)	352 (14%)	212 (4%)	212 (12%)
Don’t know	266 (5%)	-	280 (5%)	-

Numbers may not equal 100% due to rounding

### Interview data

Overall, the interviewees indicated support for these e-bike and e-scooter share schemes as they saw them as a good addition to the wider transport offer. They identified multiple potential benefits such as reduced pollution and congestion, more opportunities to access employment sites, health benefits, and journey reliability. Support was strongest for e-bike schemes. However, they felt that e-scooter schemes required more regulation, control, and enforcement. They provided a range of views on the balance between barriers to the use of these schemes and ensuring safety. These included suggestions regarding training, the need for a driving licence prior to use, and whether helmets could or should be provided to improve safety. There was the perception that these types of schemes were of good value for local authorities under financial pressure because, with the permission of the highway authority, operators owned and operated the transport service and therefore carried the financial risk. The interviewees highlighted the negative impact of privately owned e-scooters and delivery e-bikes. They indicated that the poor behaviour of some of these users affected the perception of these transport modes by others, particularly car drivers. Users of the hire schemes reported the challenges of e-bike and e-scooter use, including poor quality highway infrastructure, animosity towards e-bike and e-scooter users by other road users, and the challenge of sometimes finding points to hire or park e-bikes/e-scooters and frustration with geo-fenced areas within city centres where use is restricted. 

We asked them their opinion on how easy it was to complete the survey. In general, they indicated that it was unproblematic to complete, but found it difficult to remember because of the time elapsed since the survey (approximately 3 to 4 months). They were also asked for more details on disabilities to be included. For the resurvey questionnaire, we considered this and added ‘wheeling’ as an option whenever there were questions with transport options. However, respondents to the questionnaire used this option infrequently, with numbers too small to report separately in this paper. Vignettes for each participant, from which these summary results were derived, can be found in the Open Science repository for this project ( https://osf.io/gq9s8/, DOI 10.17605/OSF.IO/GQ9S8).

## Conclusions/discussion

The aim of this data collection was primarily for the data to be used in a future NIHR-funded evaluation of the EB and EB+ES schemes (NIHR163726; https://www.fundingawards.nihr.ac.uk/award/NIHR163726). We successfully recruited above the minimum sample size required by our power calculations in each arm for a later full evaluation. 

We found that the majority of the participants reported never having used an e-bike or an e-scooter. This is consistent with the results of the UK National Travel Survey ^1^ and Transport and Transport Technology Public Attitudes Tracker ^38^. The most common e-bike access route in the baseline sample was the use of their own personal e-bike by just under half of those who had ever used an e-bike, with access via a share-hire scheme lower by one quarter of ever users. This differed in the resurvey sample, with similar proportions reported in the overall sample for access via a share-hire scheme or their own personal e-bike. However, the most common access route reported in the control sites (Bradford and Sheffield) was personal e-bikes. In Leeds (EB), the proportions reporting these two main access routes were similar, and in the other intervention sites, the most common access route was via a share-hire scheme. This suggests that the introduction of these schemes increased people’s exposure to e-bikes as a mode of transport, as expected.

In our sample, the proportion reporting ever using an e-bike or e-scooter was higher than those who reported using these modes of transport during a ‘typical’ week. This is likely due to the proportion of the sample who tried an e-bike at least once but subsequently decided that it was not a mode of transport that they would typically use. This is plausible given outcomes from e-bike trials aimed at boosting e-bike adoption ^39, 40^. For example, the Bike4Car trial in Switzerland provided a 2-week e-bike loan in exchange for the participants’ car keys and a voucher at the end of the trial, covering ~20%–25% of the price of an e-bike to encourage purchase. High purchase cost is the most common reason for not purchasing an e-bike, according to the Transport and Transport Technology Public Attitudes Tracker ^38^. One year later, 39% said they had not purchased an e-bike and had no intention of purchasing one in the near future ^39^.

Given that e-scooter use is currently illegal outside of e-scooter trial areas in the UK ^8^, our finding that the most common e-scooter access route reported was via a share-hire scheme was unsurprising. The proportion reporting this access route in each area was also considerably higher in the e-scooter trial area than in the non-trial areas. According to the National Evaluation of e-scooter trials, the West of England Combined Authority region (which includes Bristol and Bath) had a particularly high e-scooter uptake compared to other e-scooter trial areas in the UK ^9^. However, some respondents also reported having accessed personal e-scooters, either of their own or borrowed. This was particularly high in the Bradford sample compared to the other sites. In 2022, The Parliamentary Advisory Council for Transport Safety (PACTS) collected a range of data (media reports, collision reports, and insurance claims) on private e-scooter use and estimated that approximately 750,000 private e-scooters could be in use in the UK compared with the 23,000 e-scooters available at the time via share-hire schemes ^41^. Therefore, it is not surprising that some have reported this access route.

We found that the most common destinations reported by e-bike and e-scooter users in our sample were for leisure or leisure venues, followed by work, education, shopping, and errands. A UK survey asked e-bike users (N=1112) to report their non-work use of e-bikes. Most (82%) reported using them for the ‘pleasure of the ride,’ 54% for shopping, and 43% for visiting friends/relatives ^42^. A small survey conducted in Southwest England during the Covid-19 pandemic which asked about the purpose of e-scooter users’ most recent trip, agreed with our findings, where 58% reported that their most recent trip was for socialising or leisure, followed by 22% reporting commuting for work or education and 8% for shopping and errands ^43^. The UK National Evaluation of e-scooter trials conducted during the Covid-19 pandemic in 2021 reported that while there were changes over time in reasons for e-scooter trips, in December 2021, the most popular reason for e-scooter trips was to travel to and from work (33% of trips), with leisure and personal errands, each comprising 13% of trips ^9^.

In our sample, half of the respondents said they would not use an e-bike share-hire scheme, and 63% said that they would not use an e-scooter share-hire scheme. The Transport and Transport Technology Public Attitudes Tracker ^38^ found that 10% of the respondents reported that they were likely or very likely to use an e-cycle share-hire scheme if it was available in their area. Our study found that those willing to potentially use share-hire schemes (approximately half the sample) were usually willing to walk/wheel ~500 m to access an e-bike or e-scooter. Very few people were willing to walk more than 1 km. Our results mirror a study conducted in County Dublin (N=431) that reported that the average length of time that people would be willing to walk to access an e-scooter was 4 minutes ^44^. A study of 540 participants in Zurich, Switzerland, conducted in 2020 that matched GPS tracks with micromobility app booking data ^45^, found that e-bike share-hire scheme users were willing to walk an average of 200 m up to a maximum of 490 m to access an e-bike. In contrast, e-scooter share-hire scheme users were only willing to walk 60 m on average, up to a maximum of 210 m, to access an e-scooter. We also noted this difference in our sample, with a slightly higher proportion of people willing to walk further to access an e-bike than an e-scooter.

Interviews with a diverse group of share-hire scheme users and non-users suggested overall support for share-hire schemes. They were perceived to be a good addition to the wider transport offering by increasing mobility options and helping tackle congestion and pollution from transport. This broadly agrees with previous literature, which indicates that the main reason stated for using share-hire schemes was convenience ^46, 47^. The poor quality of highway infrastructure and some frustration with the operation of schemes, including availability of hire/parking areas and restrictions on use in some city centre locations, negatively affected the experience of use. An analysis of non-users of bike and e-scooter share schemes in five European Cities supported the view that the main barriers were primarily external and infrastructural ^48^.

The purpose of this data collection was not primarily to produce representative estimates of the demographics of e-bike or e-scooter users, but to allow a specific evaluation of these schemes, which will be analysed in detail in a subsequent NIHR project (NIHR163726, https://www.fundingawards.nihr.ac.uk/award/NIHR163726). A limitation of this study is that our survey samples differed in some characteristics from the census 2021 data. However, a strength of this sample is the 1316 valid repeated measures which will be used to strengthen statistical power in the full evaluation calculations. Another limitation is the lower baseline sample size achieved by Bradford when compared to the other sites. However, we still exceeded the minimum number of responses required in each arm to satisfy our power calculations. Further, at resurvey, we achieved a similar sample size in Bradford to the other sites with the support of a local community organization. A strength of this study is the inclusion of data from two additional intervention sites for triangulation purposes. Ideally, however, the sample sizes at these two sites would have been larger, especially in Bath.

As this was a natural experiment, some external factors could not be controlled. For example, Tier, the share-hire scheme operator in Bristol and Bath, merged with Dott, which resulted in a change in the e-bike/e-scooter hiring app during the resurvey data-collection period. However, as our survey asked questions framed around ‘ever use’ and ‘typical use’ of e-bikes and e-scooters, this should have had a minimal impact on our resurvey data. Finally, with respect to generalisability, our data were collected from a limited number of sites in the UK; therefore, caution should be exercised when applying the findings more widely.

In conclusion, this study aimed to prospectively collect baseline data prior to the rollout of e-bike share-hire schemes in Bristol and Leeds and two control sites, Bradford and Sheffield. It also collected resurvey data approximately one year later at these sites and two additional share-hire scheme sites, Plymouth and Bath. These data will be used in the future in a comprehensive natural experiment evaluation of the implementation of EB and EB+ES schemes on public health, social, economic, and environmental factors.

## Data Availability

This dataset was collected for use in a full, ongoing evaluation study running from January 2025 – December 2026 (NIHR163726, https://www.fundingawards.nihr.ac.uk/award/NIHR163726), and is therefore not publicly available at this time. Following the completion of the full evaluation study, fully anonymised datasets will be made available for public reuse in 2027 via the University of Bristol online data repository (Data.bris). Please email Dr Miranda Armstrong for further information regarding data access: miranda.armstrong@bristol.ac.uk. Before sharing on Data.bris, all interview transcripts will have any possible identifying data redacted. Furthermore, all fields that could identify participants will not be shared in the dataset archived on Data.bris. Reuse is permissible under a Creative Commons Attribution 4.0 licence. The full study protocol for baseline and resurvey data collection, including the full questionnaire, can be found in the Open Science Framework online repository ( https://osf.io/gq9s8/, DOI 10.17605/OSF.IO/GQ9S8). Extended data tables referenced within this manuscript are accessible at: https://osf.io/sbg2j via the Open Science Framework (OSF): HEaLth iMpact of E-bikes and e-scooTers (HELMET): Baseline data collection for the evaluation of e-bike and e-scooter hire schemes. Doi: https://doi.org/10.17605/OSF.IO/GQ9S8 ^49^. These extended data tables are: Extended Data Table 1. Respondent characteristics at baseline Extended Data Table 2. Respondent characteristics at resurvey Extended Data Table 3. Respondent characteristics at baseline and resurvey compared with Census 2021 data Extended Data Table 4. Respondent transport use in a typical week at resurvey Data are available under the terms of the Creative Commons Attribution 4.0 International license.

## References

[1] Department for Transport: National travel survey 2023.2024. Reference Source

[2] Miner P Smith BM Jani A : Car harm: a global review of automobility's harm to people and the environment. *J Transp Geogr.* 2024;115: 103817. 10.1016/j.jtrangeo.2024.103817

[3] Philips I Anable J Chatterton T : E-bikes and their capability to reduce car CO _2_ emissions. *Transp Policy.* 2022;116:11–23. 10.1016/j.tranpol.2021.11.019

[4] Bourne JE Sauchelli S Perry R : Health benefits of electrically-assisted cycling: a systematic review. *Int J Behav Nutr Phys Act.* 2018;15(1): 116. 10.1186/s12966-018-0751-8 30463581 PMC6249962

[5] Bozzi AD Aguilera A : Shared E-scooters: a review of uses, health and environmental impacts, and policy implications of a new micro-mobility service. *Sustainability.* 2021;13(16): 8676. 10.3390/su13168676

[6] Glenn J Bluth M Christianson M : Considering the potential health impacts of electric scooters: an analysis of user reported behaviors in Provo, Utah. *Int J Environ Res Public Health.* 2020;17(17): 6344. 10.3390/ijerph17176344 32878295 PMC7503491

[7] Fishman E Cherry C : E-bikes in the mainstream: reviewing a decade of research. *Transp Rev.* 2016;36(1):72–91. 10.1080/01441647.2015.1069907

[8] Department for Transport: E-scooter trials: guidance for local areas and rental operators. London: GOV.UK,2022.

[9] NatCen Social Research and Ove Arup & Partners Ltd: National evaluation of e-scooter trials: findings report. Department for Transport,2022. Reference Source

[10] Castro A Gaupp-Berghausen M Dons E : Physical activity of electric bicycle users compared to conventional bicycle users and non-cyclists: insights based on health and transport data from an online survey in seven European cities. *Transp Res Interdiscip Perspect.* 2019;1: 100017. 10.1016/j.trip.2019.100017

[11] Sanders RL da Silva Brum-Bastos V Nelson TA : Insights from a pilot investigating the impacts of shared E-scooter use on physical activity using a single-case design methodology. *J Transp Health.* 2022;25: 101379. 10.1016/j.jth.2022.101379

[12] Pedersen BK Saltin B : Exercise as medicine–evidence for prescribing exercise as therapy in 26 different chronic diseases. *Scand J Med Sci Sports.* 2015;25(S3):1–72. 10.1111/sms.12581 26606383

[13] Ekelund U Tarp J Steene-Johannessen J : Dose-response associations between accelerometry measured physical activity and sedentary time and all cause mortality: systematic review and harmonised meta-analysis. *BMJ.* 2019;366: l4570. 10.1136/bmj.l4570 31434697 PMC6699591

[14] Foster C, the Expert Working Groups : UK Chief Medical Officers' Physical Activity Guidelines.2019. Reference Source

[15] Sport England: Active Lives adult survey, November 2022-23 Report.2024. Reference Source

[16] Sahlqvist S Goodman A Cooper AR : Change in active travel and changes in recreational and total physical activity in adults: longitudinal findings from the iConnect study. *Int J Behav Nutr Phys Act.* 2013;10(1): 28. 10.1186/1479-5868-10-28 23445724 PMC3598920

[17] Kelly P Kahlmeier S Götschi T : Systematic review and meta-analysis of reduction in all-cause mortality from walking and cycling and shape of dose response relationship. *Int J Behav Nutr Phys Act.* 2014;11(1): 132. 10.1186/s12966-014-0132-x 25344355 PMC4262114

[18] Simons M Van Es E Hendriksen I : Electrically assisted cycling: a new mode for meeting physical activity guidelines? *Med Sci Sports Exerc.* 2009;41(11):2097–102. 10.1249/MSS.0b013e3181a6aaa4 19812505

[19] Berntsen S Malnes L Langåker A : Physical activity when riding an electric assisted bicycle. *Int J Behav Nutr Phys Act.* 2017;14(1): 55. 10.1186/s12966-017-0513-z 28446180 PMC5406898

[20] Gojanovic B Welker J Iglesias K : Electric bicycles as a new active transportation modality to promote health. *Med Sci Sports Exerc.* 2011;43(11):2204–10. 10.1249/MSS.0b013e31821cbdc8 22005715

[21] Leyland LA Spencer B Beale N : The effect of cycling on cognitive function and well-being in older adults. *PLoS One.* 2019;14(2): e0211779. 10.1371/journal.pone.0211779 30785893 PMC6388745

[22] Herrmann SD Willis EA Ainsworth BE : 2024 Adult compendium of physical activities: a third update of the energy costs of human activities. *J Sport Health Sci.* 2024;13(1):6–12. 10.1016/j.jshs.2023.10.010 38242596 PMC10818145

[23] Wang K Qian X Fitch DT : What travel modes do shared e-scooters displace? A review of recent research findings. *Transp Rev.* 2023;43(1):5–31. 10.1080/01441647.2021.2015639

[24] Payne C Smith SA Sappal A : Are e-scooters active transport? Measured physical activity outputs of e-scooter riding vs walking. *J Transp Health.* 2025;41: 101963. 10.1016/j.jth.2024.101963

[25] Mathew J Liu M Seeder S : Analysis of e-scooter trips and their temporal usage patterns. *Institute of Transportation Engineers ITE Journal.* 2019;89(6):44–9. Reference Source

[26] Shaheen S Bell C Cohen A : Travel behavior: shared mobility and transportation equity.United States. Federal Highway Administration. Office of Policy …. 2017. Reference Source

[27] Grant-Muller S Yang Y Panter J : Does the use of e-scooters bring well-being outcomes for the user?: A study based on UK shared e-scooter trials. *Active Travel Studies: An Interdisciplinary Journal.* 2023;3(1). 10.16997/ats.1298

[28] Felipe-Falgas P Madrid-Lopez C Marquet O : Assessing environmental performance of micromobility using lca and self-reported modal change: the case of shared e-bikes, e-scooters, and e-mopeds in Barcelona. *Sustainability.* 2022;14(7): 4139.

[29] Bourne JE Cooper AR Kelly P : The impact of e-cycling on travel behaviour: a scoping review. *J Transp Health.* 2020;19: 100910. 10.1016/j.jth.2020.100910 32904492 PMC7456196

[30] Chang A Miranda-Moreno L Clewlow R : Trend or fad? Deciphering the enablers of micromobility in the US. *A Report of SAE International.* 2019. Reference Source

[31] Office for National Statistics: 2021 Census profile for areas in England and Wales. 2021. Reference Source

[32] Statista: Number of Facebook users in the United Kingdom (UK) from 2018 to 2027.2024. Reference Source

[33] Sammut R Griscti O Norman IJ : Strategies to improve response rates to web surveys: a literature review. *Int J Nurs Stud.* 2021;123: 104058. 10.1016/j.ijnurstu.2021.104058 34454334

[34] Mok A Khaw KT Luben R : Physical activity trajectories and mortality: population based cohort study. *BMJ.* 2019;365: l2323. 10.1136/bmj.l2323 31243014 PMC6592407

[35] Booth HP Prevost AT Gulliford MC : Epidemiology of clinical body mass index recording in an obese population in primary care: a cohort study. *J Public Health (Oxf).* 2013;35(1):67–74. 10.1093/pubmed/fds063 22829663

[36] Danquah IH Petersen CB Skov SS : Validation of the NPAQ-short – a brief questionnaire to monitor physical activity and compliance with the WHO recommendations. *BMC Public Health.* 2018;18(1): 601. 10.1186/s12889-018-5538-y 29739383 PMC5941676

[37] Ministry of Housing CLG: English indices of deprivation 2019.2019. Reference Source

[38] Department for Transport: Transport and transport technology: public attitudes tracker.London: GOV.UK, 2024.

[39] Moser C Blumer Y Hille SL : E-bike trials’ potential to promote sustained changes in car owners mobility habits. *Environ Res Lett.* 2018;13(4): 044025. 10.1088/1748-9326/aaad73

[40] Cairns S Behrendt F Raffo D : Electrically-assisted bikes: potential impacts on travel behaviour. *Transp Res Part A Policy Pract.* 2017;103:327–42. 10.1016/j.tra.2017.03.007

[41] Winchcomb M : The safety of private e-scooters in the UK. 2022. Reference Source

[42] Melia S Bartle C : Who uses e-bikes in the UK and why? *Int J Sustain Transp.* 2021;16(11):965–77. 10.1080/15568318.2021.1956027

[43] Speak A Taratula-Lyons M Clayton W : Scooter stories: user and non-user experiences of a shared e-scooter trial. *Active Travel Studies.* 2023;3(1).

[44] Carroll P : Perceptions of electric scooters prior to legalisation: a case study of Dublin, Ireland, the ‘Final Frontier’ of adopted E-Scooter use in Europe. *Sustainability.* 2022;14(18): 11376. 10.3390/su141811376

[45] Reck DJ Martin H Axhausen KW : Mode choice, substitution patterns and environmental impacts of shared and personal micro-mobility. *Transp Res D Transp Environ.* 2022;102: 103134. 10.1016/j.trd.2021.103134

[46] Sanders RL Branion-Calles M Nelson TA : To scoot or not to scoot: findings from a recent survey about the benefits and barriers of using e-scooters for riders and non-riders. *Transp Res Part A Policy Pract.* 2020;139:217–27. 10.1016/j.tra.2020.07.009

[47] Ricci M : Bike sharing: a review of evidence on impacts and processes of implementation and operation. *Res Transp Bus Manag.* 2015;15:28–38. 10.1016/j.rtbm.2015.03.003

[48] Teixeira JF Diogo V Bernát A : Barriers to bike and e-scooter sharing usage: an analysis of non-users from five European capital cities. *Case Stud Transp Policy.* 2023;13: 101045. 10.1016/j.cstp.2023.101045

[49] Armstrong MEG de Vocht F Philips I : HEaLth iMpact of E-bikes and e-scooTers (HELMET): Baseline data collection for the evaluation of e-bike and e-scooter hire schemes.NIHR13857 WP1 Extended Data Tables 1–4. [data]. OSF,2025. 10.17605/OSF.IO/GQ9S8

