# Supplementary material for: HELMET (HEaLth iMpact of E-bikes and e-scooTers) study: Data collection methods and information gathered for the evaluation of the introduction of share-hire schemes
Source: NIHR Open Res. 2025 Aug 14;5:44. Originally published 2025 May 7. [Version 2] doi: 10.3310/nihropenres.13857.2 (PMC12188184; doi:10.3310/nihropenres.13857.2)
Supplement: Peer review history

# Reviewer report: Reviewer response for version 2

**DOI:** 10.3310/nihropenres.15309.r36811

**Published:** 2025-09-08

**Authors:** James Green

**Affiliations:** 1 University of Limerick, Limerick, Ireland

I thank the authors for their thoughtful responses. The manuscript can be accepted.

Is the work clearly and accurately presented and does it cite the current literature?

Partly

If applicable, is the statistical analysis and its interpretation appropriate?

Partly

Are all the source data underlying the results available to ensure full reproducibility?

No

Is the study design appropriate and is the work technically sound?

Partly

Are the conclusions drawn adequately supported by the results?

Partly

Are sufficient details of methods and analysis provided to allow replication by others?

Yes

Reviewer Expertise:

modal shift, sustainable mobility, health psychology, quantitative methods

I confirm that I have read this submission and believe that I have an appropriate level of expertise to confirm that it is of an acceptable scientific standard.

# Reviewer report: Reviewer response for version 1

**DOI:** 10.3310/nihropenres.15058.r35720

**Published:** 2025-06-28

**Authors:** James Green

**Affiliations:** 1 University of Limerick, Limerick, Ireland

Overall, this paper presents a useful insight into users and non-users of ebikes and e-scooters, both through private ownership and use of shared schemes. I have some recommendations which I think would help more clearly focus this paper.

 Being clearer about the study aims and objectives here would be an important modification. This should include the overlap with your other planned work, as well as external sources such as the Walking and Cycling Index. Clarify the relationship between the pre-registration document and the data presented here. From my reading, it looks like the pre-registration was for baseline only, and the results presented here include the resurvey data. And then the further addition of the Plymouth and Bath as triangulation cities.

 This should also make it clearer why the recruitment goal was to enhance the response rate, as opposed to ensuring representativeness. It seems that a large response rate is important for statistical power for the planned analysis of data, whereas this seems like it probably decreases the representativeness of the data for the purposes of the present presentation.

 Following on from this, given that the present data is primarily descriptive, consideration could be given to population-weighting survey responses, though given the fairly extreme deviations (eg in terms of education in the sample), this would need to be done carefully.

 As the primary results are presented here in the form of percentages, while it is possible to compute 95% confidence intervals for each percentage estimate, it might clearer to calculate the margin of error for each sample, to illustrate precision. By convention, this is usually done for an estimate of 50% (which also corresponds to the largest margin of error), eg for Bradford at baseline, 1.96 x (.5*(1-5))/sqrt(521) = +/- 4.29%. This can then provide a rough estimate as to whether differences between cities, baseline vs re-survey etc. are of a large enough magnitude to be a reliable difference.

 Overall, there is a lot of tabular data, which is relatively hard to interpret for the reader, with for example baseline and resurvey often in different tables, and then the data about the overall sample vs e-scooter and e-bike users presented separately. So if a reader wanted to consider whether those using micro-mobility were more or less likely to have a health condition, this comparison would be challenging. Perhaps moving more tables to the extended data table section, and presenting some summary graphs that enable more comparisons to be visualised could be a good solution to this.

 It would also be important to revisit the limitations of the sampling, and the different aims of the future work vs. current in the discussion.

 More minor points

  

1. Anecdotally, it seems that there is a link between transport poverty and the use of personally-owned e-scooters. (For example, I see large numbers of e-scooters before buses start running in the morning, presumably enabling people to get to shift work). Considering if there is an association between SES and private scooter use would therefore be very interesting, esp. given high rates in Bradford.
2. On p.16, there is reference to ‘County Dublin’. While the authors may have described their area as County Dublin, this would be clearer as just ‘Dublin’ for an international audience. (Briefly, County Dublin no longer exists, but is short-hand for the 4 authorities that make up ‘Dublin’, but is what most people would think of corresponding to Dublin).
3. P.14 – refs 39-40 are both relatively old now, and there is a considerable amount of more recent research that could be included here.

Is the work clearly and accurately presented and does it cite the current literature?

Partly

If applicable, is the statistical analysis and its interpretation appropriate?

Partly

Are all the source data underlying the results available to ensure full reproducibility?

No

Is the study design appropriate and is the work technically sound?

Partly

Are the conclusions drawn adequately supported by the results?

Partly

Are sufficient details of methods and analysis provided to allow replication by others?

Yes

Reviewer Expertise:

modal shift, sustainable mobility, health psychology, quantitative methods

I confirm that I have read this submission and believe that I have an appropriate level of expertise to confirm that it is of an acceptable scientific standard, however I have significant reservations, as outlined above.

# Response

**Published:** 2025-08-07

**Authors:** James Garbutt

**Affiliations:** University of Bristol Centre for Sport Exercise and Health, Bristol, England, UK

Comments from and responses to Reviewer #3:

 Overall, this paper presents a useful insight into users and non-users of ebikes and e-scooters, both through private ownership and use of shared schemes. I have some recommendations which I think would help more clearly focus this paper.

 Being clearer about the study aims and objectives here would be an important modification. This should include the overlap with your other planned work, as well as external sources such as the Walking and Cycling Index. Clarify the relationship between the pre-registration document and the data presented here. From my reading, it looks like the pre-registration was for baseline only, and the results presented here include the resurvey data. And then the further addition of the Plymouth and Bath as triangulation cities.

**Response 1**: We would like to thank reviewer 3 for taking the time to review our article and for the helpful comments they provided. We now realise that we had not specifically linked to the updated data collection protocol document, only the overall project registration page (DOI 10.17605/OSF.IO/GQ9S8). For background, funding for resurvey data collection was granted after baseline data collection had begun, meaning there exists both a baseline data collection protocol and an amended baseline plus resurvey data collection protocol within the OSF project pages. This quite rightly may have caused some confusion. We have now updated URL’s when the protocol is referenced to direct readers directly to the protocol covering both baseline and resurvey data collection (https://osf.io/8ywps).

 In regards to making the aims of the manuscript more explicit, several text amendments have been made (see below, highlighted in bold). However, the only aim of this manuscript was to provide the summary descriptives of data that will subsequently be analysed within the full scheme evaluation project (which we have linked to within the manuscript but understandably not described in great detail as this is not the aim of this preliminary data descriptives paper):

 Abstract: “Background This study aimed to collect **and summarise** information on e-bike and e-scooter use in areas with and without e-bike (EB) and e-bike plus e-scooter (EB+ES) combined share-hire schemes.”

 Last paragraph of introduction: “ **Thus, while it is evident that e-bike (EB) and combined e-bike/e-scooter (EB+ES) share hire schemes could impact on public health, social, economic, and environmental factors, numerous research gaps still remain.** This study aimed to collect information on e-bike and e-scooter use in areas with and without EB and EB+ES combined share-hire schemes **and summarise this information descriptively in preparation for a future, full** evaluation of the impacts of EB and EB+ES share-hire schemes on public health, social, economic, and environmental factors (NIHR163726; https://www.fundingawards.nihr.ac.uk/award/NIHR163726). **Specifically, we aimed to collect information pre- and post-EB share-hire scheme introduction on demographics, physical activity volumes, use of e-bikes or e-scooters, active travel and other travel, distance travelled by different transport modes, substitution of travel modes, quality of life, and access to venues and services.**”

 Study design and setting: “…The main project included four major English cities, with baseline and resurvey data. Leeds had no share-hire scheme at baseline, but launched an e-bike share-hire scheme (EB) in mid-September 2023 (baseline data collection was stopped the day before the launch of the scheme). Bristol had an e-scooter share-hire scheme only at baseline but launched a combined e-bike and e-scooter share-hire scheme (EB+ES) in October 2023. Bradford and Sheffield were non-intervention control areas without share-hire schemes. **This will provide three levels of exposure (EB only, EB+ES combined, no such schemes) for exploring health, social, economic, and environmental impacts in the subsequent full EB and/or ES share-hire scheme evaluation**. …”

 This should also make it clearer why the recruitment goal was to enhance the response rate, as opposed to ensuring representativeness. It seems that a large response rate is important for statistical power for the planned analysis of data, whereas this seems like it probably decreases the representativeness of the data for the purposes of the present presentation. Following on from this, given that the present data is primarily descriptive, consideration could be given to population-weighting survey responses, though given the fairly extreme deviations (eg in terms of education in the sample), this would need to be done carefully.

**Response 2**: We agree that we sought a large enough response rate for achieving sufficient analysis power based on our minimum sample size calculation. We also acknowledge that online surveys collect only voluntary responses and do not typically achieve population-representative samples. In line with this, we have rightly added in further detail on the latter as a limitation within the discussion (additions highlighted in bold):

 “The purpose of this data collection was not primarily to produce representative estimates of the demographics of e-bike or e-scooter users, but to allow a specific evaluation of these schemes, which will be analysed in detail in a subsequent NIHR project (NIHR163726, https://www.fundingawards.nihr.ac.uk/award/NIHR163726). **We did not apply population weightings to the data for this reason. In line with this, a** limitation of this study is that our survey samples differed in some characteristics from the census 2021 data. **Specifically, our sample was generally skewed towards those of white ethnicity who were older and had higher education levels. Data was collected online using non-probability (i.e. voluntary response) convenience sampling and self-selection bias is a known limitation of such surveys (Groves, Fowler Jr et al. 2011). Survey access was also largely restricted to those with internet access except where responses were facilitated in-person by local community advocates.**”

 Although we could never guarantee sample representativeness due to the online survey method used, the additional methods for data collection we employed at resurvey in Bradford were specifically aimed at maximising this for our data for the Bradford population. Given what we knew about the greater ethnic diversity in Bradford versus the other cities, it was reasonable to conclude that the lower response rate at baseline was likely low due to insufficient public reach to harder to reach voices. As stated within the ‘Resurvey survey data collection’ section, we sought to capture this population diversity by working with local charity and community organisations to maximise reach and, in turn, overcome the lower total response rate observed at baseline. In general, we have no reason to believe that achieving a larger sample size in any of the cities would necessarily be a cause of reduced sample representativeness. We believe the limitations inherent to online surveys would be the over-arching reason for this.

 As stated, we plan to use the collected data within a subsequent full analysis and evaluation. Given the known limitations of online surveys and their inability to achieve truly representative samples, we have not applied population weightings to the data as this may potentially give a false sense of confidence in the summary data reported to readers. Practically, the reviewer is also correct to highlight that education is highly skewed and therefore the application of weightings would have needed approaching carefully.

 As the primary results are presented here in the form of percentages, while it is possible to compute 95% confidence intervals for each percentage estimate, it might clearer to calculate the margin of error for each sample, to illustrate precision. By convention, this is usually done for an estimate of 50% (which also corresponds to the largest margin of error), eg for Bradford at baseline, 1.96 x (.5*(1-5))/sqrt(521) = +/- 4.29%. This can then provide a rough estimate as to whether differences between cities, baseline vs re-survey etc. are of a large enough magnitude to be a reliable difference.

**Response 3**: Confidence intervals and margins of error are inferential statistics which we did not want to present in this paper as this was not it’s overall purpose. Our aim was to provide descriptives only and this is stated within the paper and also in a previous review response. We will be looking at differences between cities and timepoints using a more robust model in our later analyses/scheme evaluation. It is not customary to add percentage margins of error but as you rightly demonstrated, this can be calculated by readers from the data provided. However, we purposely do not want to encourage readers to calculate this based on the raw data prior to our conducting more robust analyses.

 Overall, there is a lot of tabular data, which is relatively hard to interpret for the reader, with for example baseline and resurvey often in different tables, and then the data about the overall sample vs e-scooter and e-bike users presented separately. So if a reader wanted to consider whether those using micro-mobility were more or less likely to have a health condition, this comparison would be challenging. Perhaps moving more tables to the extended data table section, and presenting some summary graphs that enable more comparisons to be visualised could be a good solution to this.

**Response 4**: We agree with the reviewer that the manuscript contained a lot of tabular data. As suggested, we have now halved the number of tables within the main manuscript from 10 to 5 (moving tables 1-5 to Extended data) and also presented the key gender and age characteristics of the baseline and resurvey samples for Bradford, Bristol, Leeds and Sheffield combined graphically instead (Figure 2). The text referring to each has been updated accordingly.

 It would also be important to revisit the limitations of the sampling, and the different aims of the future work vs. current in the discussion.

**Response 5**: We have now added additional limitations within the discussion as highlighted in our previous response, as well as noting the aim of the current study was for data collection only (copied again below).

 “The purpose of this data collection was not primarily to produce representative estimates of the demographics of e-bike or e-scooter users, but to allow a specific evaluation of these schemes, which will be analysed in detail in a subsequent NIHR project (NIHR163726, https://www.fundingawards.nihr.ac.uk/award/NIHR163726). **We did not apply population weightings to the data for this reason.** In line with this, a limitation of this study is that our survey samples differed in some characteristics from the census 2021 data. **Specifically, our sample was generally skewed towards those of white ethnicity who were older and had higher education levels. Data was collected online using non-probability (i.e. voluntary response) convenience sampling and self-selection bias is a known limitation of such surveys (Groves, Fowler Jr et al. 2011). Survey access was also largely restricted to those with internet access except where responses were facilitated in-person by local community advocates.**”

 Within the final paragraph of the discussion, we have also now expanded on what we aim to do with the data within the full evaluation:

 “… **Utilising these data under the framework of a natural experiment evaluation, we will determine the impact of such schemes on group-level physical activity and travel mode changes (modal shifts), and calculate the health economics and potential carbon savings under such schemes; crucial information for guiding policymakers on what benefits introducing such schemes might have on public health, society and the environment.**”

 More minor points

- Anecdotally, it seems that there is a link between transport poverty and the use of personally-owned e-scooters. (For example, I see large numbers of e-scooters before buses start running in the morning, presumably enabling people to get to shift work). Considering if there is an association between SES and private scooter use would therefore be very interesting, esp. given high rates in Bradford.

**Response 6**: We agree that this would definitely be an interesting area to investigate further but we don’t believe this dataset would be best suited to that. This is due to private e-scooter use not being the focus of our intended work and due to their illegality outside of government-approved scheme areas, meaning respondents may not have answered truthfully about private e-scooter use. We would not consider the current dataset with its focus on public share-hire schemes being of sufficient quality to investigate private e-scooter use in detail.

  

- On p.16, there is reference to ‘County Dublin’. While the authors may have described their area as County Dublin, this would be clearer as just ‘Dublin’ for an international audience. (Briefly, County Dublin no longer exists, but is short-hand for the 4 authorities that make up ‘Dublin’, but is what most people would think of corresponding to Dublin).

**Response 7**: Thank you for this clarification. We have now corrected ‘County Dublin’ to just ‘Dublin’.

  

- P.14 – refs 39-40 are both relatively old now, and there is a considerable amount of more recent research that could be included here.

**Response 8**: We have included additional references here that are more recent. The text now reads as follows (additions highlighted in bold):

**“**In our sample, the proportion reporting ever using an e-bike or e-scooter was higher than those who reported using these modes of transport during a ‘typical’ week. This is likely due to the proportion of the sample who tried an e-bike at least once but subsequently decided that it was not a mode of transport that they would typically use. This is plausible given outcomes from e-bike trials aimed at boosting e-bike adoption, **which indicate that short-term access encourages initial uptake and increases purchase intentions, but** **long-term adoption remains limited ^39^^, ^^40^(Department for Transport & Steer 2021, Ton and Duives 2021, Wilson and Whitmarsh 2025).** **For example, the national e-cycle programme reported that only 7% of participants purchased an e-bike following a one-month loan, despite 72% expressing greater willingness to do so (Department for Transport & Steer 2021).** **A more recent field study reported that although e-bike loans reduced car dependency and increased cycling frequency, just 29% of participants went on to purchase an e-bike (Wilson and Whitmarsh 2025). T**he Bike4Car trial in Switzerland provided a 2-week e-bike loan in exchange for the participants’ car keys and a voucher at the end of the trial, covering ~20%–25% of the price of an e-bike to encourage purchase (Moser, Blumer et al. 2018). **One year later, 39% said they had not purchased an e-bike and had no intention of purchasing one in the near future ^39 ^.** High purchase cost is the most common reason for not purchasing an e-bike, according to the **2024** Transport and Transport Technology Public Attitudes Tracker ^38^ .”

# Reviewer report: Reviewer response for version 1

**DOI:** 10.3310/nihropenres.15058.r35722

**Published:** 2025-06-24

**Authors:** Jenna McVicar

**Affiliations:** 1 Monash University Faculty of Medicine Nursing and Health Sciences, Clayton, Victoria, Australia

Dear editor,

 Thank you for allowing me the opportunity to review this manuscript. It is extremely well written and a thoughtful piece of work and would recommend acceptance of the article. 

 Here are some comments:

 This is a well-designed study and methods are explained appropriately to allow for replication. Additional links provided to protocol registration and full questionnaire are extremely helpful. 

 Statistical analysis and interpretation are appropriate for study design. 

 Source data is available for the interviews and link provided, however authors state “This dataset was collected for use in a full, ongoing evaluation study running from January 2025 – December 2026 (NIHR163726, https://www.fundingawards.nihr.ac.uk/award/NIHR163726), and is therefore not publicly available at this time”.

 Conclusion/discussion provides understanding of the results within current literature. These findings add to the understanding and peoples perceptions of e-bikes and e-scooters in relation to hire schemes.

Is the work clearly and accurately presented and does it cite the current literature?

Yes

If applicable, is the statistical analysis and its interpretation appropriate?

Yes

Are all the source data underlying the results available to ensure full reproducibility?

Partly

Is the study design appropriate and is the work technically sound?

Yes

Are the conclusions drawn adequately supported by the results?

Yes

Are sufficient details of methods and analysis provided to allow replication by others?

Yes

Reviewer Expertise:

Physical activity, exercise physiology, health & behaviour change.

I confirm that I have read this submission and believe that I have an appropriate level of expertise to confirm that it is of an acceptable scientific standard.

# Response

**Published:** 2025-08-07

**Authors:** James Garbutt

**Affiliations:** University of Bristol Centre for Sport Exercise and Health, Bristol, England, UK

We would like to thank reviewer 2 for taking the time to review the article and for their positive comments.

# Reviewer report: Reviewer response for version 1

**DOI:** 10.3310/nihropenres.15058.r35584

**Published:** 2025-05-30

**Authors:** Helaine Alessio

**Affiliations:** 1 Miami University, Oxford, Ohio, USA

General Comments:

 The HELMET study used a repeated cross-sectional design with an online survey gathering data about demographics, travel and health for 2 months one year before ebike and escooter sharing programs were launched in two cities (one implemented both ebikes and escooters and another just ebikes) and used two different cities with no ebike or escooter sharing programs as a control. A re-survey was conducted a year later, and added two more cities. Also--8 ebike and escooter users were interviewed for qualitative information.

 The aim of this study was to collect data on ebike and escooter use in areas with and without ebike and escooter sharing programs to evaluate ebike and escooter share programs to be used in a future grant application. 

 The authors were successful in obtaining data from 3771 surveys (I think they were complete or at least acceptable) and 5370 in the resurvey--the higher number was due to the two additional cities that were surveyed in the second year.

 The information obtained clearly showed more interest and participation in ebike vs. escooter riding. They also showed that most of the people who participated in this survey met or exceeded physical activity guidelines, and the clear majority (over 67%) reported no health conditions. So, the population was a relatively young (the majority were under age 44 years old and were healthy and active. No surprises there but important to affirm to whom these programs are marketed. As for destinations and access distances, the authors reported that most ebike users rode their own ebikes and most escooter riders used their own or borrowed one from someone. Half of the ebike riders claimed they would not use an ebike share-hire scheme and 63% indicated they would not use an escooter share hire scheme. Nevertheless, the 8 individuals interveiwed indicated support for ebike and escooter share schemes and saw them as a good addition to the wider transportation support options

 I think that the data presented was interesting and valuable, however, the authors could have been a little more specific about what types of specific requests or plans the data implied instead of just stating that they would use the data in a future grant proposal.  The authors left it to the reader to imagine what specific information did the authors obtain from these surveys and interviews that would guide them towards a specific "ask" in a future grant proposal.

 It seems that trends from the surveys included age (most ebike/scooter riders were relatively young adults) and reason for travel (mostly for leisure) should have been highlighted more. It may be that this information (young adults prefering to ride ebikes for leisure travel) would be helpful in planning dedicated trails for leisure--for example--that would directly relate to the interest in those surveyed.  This info would be important for future planning. 

 Specific Comments

 Page 1

 Methods section- How were surveys distributed to people? How were the people contacted? Was there a list of residents? If surveys were sent out--how many were completed--what was the percent return/completion?

 Page 4

 Methods - Patient and Public Involvement -- why use the term "patient" in the methods when the survey takers appeared to be community dwelling healthy adults?

 Page 5 

 Baseline survey data collection-

 Where were the advertisements posted? Local newspapers? And what mailing lists were used?

 It is not clear who the following are:  "...relevant local stakeholder groups and gatekeepers of relevant social media groups in each study area..."  Please identify these people.

 Page 7

 Results - 

 "At baseline 4271 online surveys were completed."  Were they all complete? Or were they submitted and some or most were complete?

 Same question a few lines later where it is stated that "6160 online surveys were completed." Were they completed or submitted?

 Conclusion-

 The concluding paragraph starting with "In conclusion, this study aimed to prospectively collect baseline data...." really doesn't provide conclusions from the surveys. 

 It just indicated that the data will be used in a future experiment.

 This needs to be addressed.  The concluding paragraph should summarize and succinctly state some major conclusions associated with the data that was collected.

Is the work clearly and accurately presented and does it cite the current literature?

Yes

If applicable, is the statistical analysis and its interpretation appropriate?

Yes

Are all the source data underlying the results available to ensure full reproducibility?

Yes

Is the study design appropriate and is the work technically sound?

Yes

Are the conclusions drawn adequately supported by the results?

Partly

Are sufficient details of methods and analysis provided to allow replication by others?

Yes

Reviewer Expertise:

Kinesiology, Public Health

I confirm that I have read this submission and believe that I have an appropriate level of expertise to confirm that it is of an acceptable scientific standard.

# Response

**Published:** 2025-08-07

**Authors:** James Garbutt

**Affiliations:** University of Bristol Centre for Sport Exercise and Health, Bristol, England, UK

Comments from and responses to Reviewer #1:

 I think that the data presented was interesting and valuable, however, the authors could have been a little more specific about what types of specific requests or plans the data implied instead of just stating that they would use the data in a future grant proposal.  The authors left it to the reader to imagine what specific information did the authors obtain from these surveys and interviews that would guide them towards a specific "ask" in a future grant proposal.

**Response 1**: We would like to thank reviewer 1 for taking the time to review our article and for the helpful comments provided to improve the manuscript. We have sought to address the concerns raised, including refining the concluding paragraph of the study and likewise, added in brief descriptions of our intentions of what we intend to analyse from the data (changes in reported physical activity, transport mode use (modal shifts) as well as calculating health economics and potential carbon savings). The concluding paragraph now reads as (modified text in bold):

               “In conclusion, **the HELMET baseline and resurvey data provides a detailed picture of public access to and use of e-bikes and e-scooters within six cities in England, achieving the required sample sizes for conducting a future full scheme evaluation. Most people surveyed had never used an e-bike or e-scooter, with a mixed willingness to try using them in future, indicating there remains considerable untapped potential for increasing e-transport use within the general population, provided there is appropriate implementation and infrastructure. Although most e-bike and e-scooter use was reported as being for leisure, users reported a variety of travel purposes, including commuting to work and/or school, suggesting higher public use could increase active transport both recreationally and functionally. Access methods to e-bikes and e-scooters differed by vehicle type and location, largely in accordance with public share-hire availability and, in turn, the legal status of e-scooter use outside of such areas. Utilising these data under the framework of a natural experiment evaluation, we will determine the impact of such schemes on group-level physical activity and travel mode changes (modal shifts), and calculate the health economics and potential carbon savings under such schemes; crucial information for guiding policymakers on what benefits introducing such schemes might have on public health, society and the environment.**”

 2. It seems that trends from the surveys included age (most ebike/scooter riders were relatively young adults) and reason for travel (mostly for leisure) should have been highlighted more. It may be that this information (young adults prefering to ride ebikes for leisure travel) would be helpful in planning dedicated trails for leisure--for example--that would directly relate to the interest in those surveyed.  This info would be important for future planning.

**Response 2**: Due to the use of convenience sampling, our study wasn't designed to provide representative estimates for e-bike or e-scooter users in the overall population. We highlight this within paragraph 8 of the discussion and also have expanded on limitations of the dataset for making immediate inference:

 “The purpose of this data collection was not primarily to produce representative estimates of the demographics of e-bike or e-scooter users, but to allow a specific evaluation of these schemes, which will be analysed in detail in a subsequent NIHR project (NIHR163726, https://www.fundingawards.nihr.ac.uk/award/NIHR163726). **We did not apply population weightings to the data for this reason. In line with this**, a limitation of this study is that our survey samples differed in some characteristics from the census 2021 data. **Specifically, our sample was generally skewed towards those of white ethnicity who were older and had higher education levels. Data was collected online using non-probability (i.e. voluntary response) convenience sampling and self-selection bias is a known limitation of such surveys (Groves, Fowler Jr et al. 2011). Survey access was also largely restricted to those with internet access except where responses were facilitated in-person by local community advocates.** …”

 For your example, although the number of adults who reported ever using an e-bike or e-scooter was greater in younger age groups overall, and the most common purpose for using an e-bike or e-scooter in the sample overall was for leisure, we haven’t evidenced that young adults prefer to ride e-bikes for leisure travel within this manuscript. Given the intention of a full evaluation, drawing conclusions for informing potential policy at this stage of analysis was not the aim of this manuscript. Policy implications will however be a focus within the wider NIHR HELMET evaluation/analysis (ongoing), as highlighted in the concluding paragraph, given we will be undertaking more robust analyses at that point.

 3. Specific Comments

 Page 1

 Methods section- How were surveys distributed to people? How were the people contacted? Was there a list of residents? If surveys were sent out--how many were completed--what was the percent return/completion?

**Response 3:** Thank you for your feedback here. We have improved clarity of how the survey was hosted online, distributed and to whom via the following edits (highlighted in yellow):

 “Online survey. The baseline survey and resurvey, developed in consultation with council communication and transport teams from the four local authorities, contained 32 questions for the baseline survey and 41 questions for the resurvey. The full questionnaire information can be found in the protocol ( https://osf.io/gq9s8/, DOI 10.17605/OSF.IO/GQ9S8). **The baseline survey was hosted by the JISC ‘Online Surveys’ platform (https://www.onlinesurveys.ac.uk) and the resurvey was hosted by the Qualtrics platform (https://www.qualtrics.com). Qualtrics was used at resurvey due to a loss of required functionality on JISC Online Surveys due to platform revisions taking place at the time of resurvey data collection. Each survey** inquired about demographics …”

             “ *Baseline Survey Data Collection.* Baseline recruitment **used convenience sampling to target** residents aged 16 years and over of the two intervention local authorities, Leeds and Bristol, and the two control local authorities, Sheffield and Bradford. The intervention and control sites were similar with respect to the proportion reporting very good health (50%, 51%, 47%, 46%) and income deprivation (14%, 14%, 16%, 19%) ^31^ .

 The primary mode of distribution for our baseline survey was through advertisements with a **web**link to our survey via council-administered **electronic** newsletters and/or mailing lists **(councils provided contact information of local “council-friendly” organisations for inviting directly by researchers)** and council-administered social media **on Facebook and X/Twitter. A mailing contact list was unavailable for Bradford. Therefore, researchers created one manually using DivaBradford.org.uk, a local online directory. All mailing list organisations were assessed for suitability for study inclusion through inspecting their website and excluding those where the client base were considered potentially unable to read or write English, were experiencing addiction or domestic violence, or were under the age of 16.** We supplemented **council-based distribution routes** with secondary methods. First, paid-for Facebook advertising, which has extensive reach with 79% of UK adults who go online using Facebook and 45% using it on a daily basis ^32^ . Second, **additional** local stakeholder groups **(including cycling clubs and active travel interest groups, ethnic minority related groups, local community forums, housing associations, charities and community organisations, local businesses and gatekeepers to posting on** relevant social media groups in each study area **on Facebook and Twitter/X) were identified by researchers** for facilitated distribution **of survey weblinks** to their **staff/**members, improving reach to seldom heard voices. Third, we used a prize draw in each region as an incentive, as including entry into prize draws has been shown to improve response rates to surveys ^33^ .”

 “…As Bath had a much smaller population size than the other sites, we also supplied physical posters containing a QR code link to the online survey in public places **(e.g., bus stops, supermarkets, libraries, community notice boards)** to increase survey reach.”

 4: Page 4

 Methods - Patient and Public Involvement -- why use the term "patient" in the methods when the survey takers appeared to be community dwelling healthy adults?

**Response 4:** “Patient and Public Involvement” is a catch-all term used by the NIHR related to patient or public engagement in the research design stage. Although it is a standardised term, we have added quotation marks to the term within the section text in an attempt to highlight this.

               “… We also had a **“**Patient and Public Involvement **”** (PPI) member embedded in the research team who provided important input during research team meetings and advised on documentation, especially that which was publicly facing.”

 5. Page 5

 Baseline survey data collection-

 Where were the advertisements posted? Local newspapers? And what mailing lists were used?

**Response 5**: Please see response number 3.

 6. It is not clear who the following are:  "...relevant local stakeholder groups and gatekeepers of relevant social media groups in each study area..."  Please identify these people.

**Response 6**: Please see response number 3. We have summarised the types of groups/stakeholders we contacted for distribution of the surveys. We cannot identify these further due to GDPR rules.

 7. Page 7

 Results -

 "At baseline 4271 online surveys were completed."  Were they all complete? Or were they submitted and some or most were complete?

 Same question a few lines later where it is stated that "6160 online surveys were completed." Were they completed or submitted?

**Response 7**: We have now edited the text appropriately to read ‘submitted’ rather than completed. The section now reads:

               “At baseline, 4271 online surveys were **submitted**. Following data cleaning and duplicate removal, N=3771 (n=1048 for Bristol, n=1096 for Leeds, n=521 for Bradford, and n=1106 for Sheffield) remained for the baseline data analysis. In the resurvey, 6160 online surveys were **submitted**. After data cleaning and duplicate removal, N=5370 (n=1153 for Bristol, n=1192 for Leeds, n=1006 for Bradford, n=1024 for Sheffield, n=363 for Bath, and n=632 for Plymouth) remained for the resurvey data analysis.

 8. Conclusion-

 The concluding paragraph starting with "In conclusion, this study aimed to prospectively collect baseline data...." really doesn't provide conclusions from the surveys.

 It just indicated that the data will be used in a future experiment.

 This needs to be addressed.  The concluding paragraph should summarize and succinctly state some major conclusions associated with the data that was collected.

**Response 8**: Please see the edited concluding paragraph shown in response number 1.
